# Molecular Evolution of Transforming Growth Factor-β (TGF-β) Gene Family and the Functional Characterization of Lamprey TGF-β2

**DOI:** 10.3389/fimmu.2022.836226

**Published:** 2022-03-04

**Authors:** Siqi Liu, Junfu Guo, Xianda Cheng, Wenna Li, Shuangyu Lyu, Xuanyi Chen, Qingwei Li, Hao Wang

**Affiliations:** ^1^ College of Life Sciences, Liaoning Normal University, Dalian, China; ^2^ Lamprey Research Center, Liaoning Normal University, Dalian, China; ^3^ Collaborative Innovation Center of Seafood Deep Processing, Dalian Polytechnic University, Dalian, China

**Keywords:** TGF-β, cytokine, lamprey, phylogeny, immune system

## Abstract

The transforming growth factor-βs (TGF-βs) are multifunctional cytokines capable of regulating a wide range of cellular behaviors and play a key role in maintaining the homeostasis of the immune system. The TGF-β subfamily, which is only present in deuterostomes, expands from a single gene in invertebrates to multiple members in jawed vertebrates. However, the evolutionary processes of the TGF-β subfamily in vertebrates still lack sufficient elucidation. In this study, the TGF-β homologs are identified at the genome-wide level in the reissner lamprey (*Lethenteron reissneri*), the sea lamprey (*Petromyzon marinus*), and the Japanese lamprey (*Lampetra japonica*), which are the extant representatives of jawless vertebrates with a history of more than 350 million years. The molecular evolutionary analyses reveal that the lamprey TGF-β subfamily contains two members representing ancestors of TGF-β2 and 3 in vertebrates, respectively, but TGF-β1 is absent. The transcriptional expression patterns show that the lamprey TGF-β2 may play a central regulatory role in the innate immune response of the lamprey since it exhibits a more rapid and significant upregulation of expression than TGF-β3 during lipopolysaccharide stimuli. The incorporation of BrdU assay reveals that the lamprey TGF-β2 recombinant protein exerts the bipolar regulation on the proliferation of the supraneural myeloid body cells (SMB cells) in the quiescent and LPS-activated state, while plays an inhibitory role in the proliferation of quiescent and activated leukocytes in lampreys. Furthermore, caspase-3/7 activity analysis indicates that the lamprey TGF-β2 protects SMB cells from apoptosis after serum deprivation, in contrast to promoting apoptosis of leukocytes. Our composite results offer valuable clues to the origin and evolution of the TGF-β subfamily and imply that TGF-βs are among the most ancestral immune regulators in vertebrates.

## Introduction

The transforming growth factor-β (TGF-β) superfamily is a large family of multifunctional cytokines containing many members. More than 60 TGF-β superfamily members have been identified in multicellular organisms and can be classified into TGF-βs, Activins, Inhibins, bone morphogenetic proteins (BMPs), growth, and differentiation factors (GDFs), Nodals, Myostatins, MIS (Müllerian-inhibiting substance), and Lefty. The TGF-β superfamily signaling pathway, one of the fundamental and versatile signal transduction pathway in metazoans, plays a crucial role in the development of organisms and the maintenance of tissue homeostasis (1). Members of the TGF-β superfamily have emerged since the early evolution of the animal kingdom, and conserved members of the TGF-β pathway are present in all metazoans studied (2). The first members of the TGF-β superfamily to emerge were BMPs/GDFs, which subsequently differentiated into Activins/Inhibins, while the ‘ture’ TGF-β subfamily emerged later and is only present in deuterostomes (3, 4). Both the two lower phyla of deuterostomes, including sea urchins (Echinodermata) and acorn worms (Hemichordata), contain a single true TGF-β homolog, while sea squirts (Urochordata) and amphioxus (Cephalochordata) in the Chordata also contain a single TGF-β homolog. In jawed vertebrates, the TGF-β subfamily includes mainly three members, TGF-β1~3, with high amino acid sequence homology (5). The expansion of family members probably originated from the duplication of a common ancestral gene and was widely dispersed through chromosomal translocations (6). However, the origin and evolution of all members of the TGF-β subfamily in other representative species have remained unclear to date due to a lack of high-quality genomic data, except for studies in humans and other model animals. Recently, high-quality sequencing of the genome of one of the extant representatives of the jawless vertebrates, the reissner lamprey (*Lethenteron reissneri*), has been completed (7), offering new possibilities for filling key links in the evolution of TGF-βs.

The precursor protein encoded by the TGF-βs gene consists of an N-terminal signal peptide, a prodomain, and a C-terminal mature peptide domain (ligand domain), which contains a “cystine knot” of nine conserved cysteines that are required for the folding and dimerization of the biologically active TGF-βs ligand. The processing of TGF-βs precursor proteins is a complex and tightly regulated process. Within the endoplasmic reticulum, TGF-βs precursors form dimeric complexes that are subsequently cleaved and processed into mature TGF-β homodimers by the furin in the trans-Golgi network, while the released N-terminal propeptides form latent associated peptide (LAP) dimers (8). LAP binds TGF-β with high affinity through non-covalent bonds to form a small latent complex (SLC) in which the receptor-binding site of the TGF-β ligand is shielded from binding to its cell surface receptor by LAP (9). Additionally, a latent TGF-β binding protein (LTBP) in the ER binds the SLC complex of TGF-β through a disulfide bond, which is formed between the 33rd cysteine residues in each of the two LAP chains and a pair of cysteine residues in LTBP (10, 11). The combination of LTBP and SLC is known as a large latent complex (LLC), which anchors TGF-β into the extracellular matrix *via* LTBP. The triggering of TGF-β signaling is not dependent on the secretion of TGF-β ligands from cells, but on the release of mature TGF-β homodimers from the latent complex by latent TGF-β activators (e.g., cell surface integrins αvβ6 and αvβ8). Physiological activation of this latent complex is a key regulatory step in the control of TGF-β bioactivity (12).

Mature TGF-β dimers activate signal transduction through a transmembrane serine/threonine kinase receptor complex, including type I receptors and type II receptors. Type II receptors can bind to TGF-β ligands with high affinity and subsequently phosphorylate type I receptors, leading to autophosphorylation of type I receptors and phosphorylation of downstream receptor-activated Smads (R-Smads), i.e., Smad2 and 3. Subsequently, every two R-Smads form a heterotrimer with a Co-Smad (i.e., Smad4) that translocates into the nucleus, and the R-Smads-Smad4 complex directly recruits multiple transcriptional co-activators or co-repressors and binds to the promoter of its target gene, thereby activating or repressing the expression of the target gene. TGF-βs, as multifunctional cytokines, can regulate proliferation, differentiation, adhesion, migration, and apoptosis at the cellular level, thus maintaining the homeostasis of embryonic development and adult systems under normal physiological conditions. For example, stimulation with TGF-βs induces cytostasis in almost all mammalian cells, such as epithelial cells, endothelial cells, hematopoietic cells, and neurons, yet can promote the proliferation of renal fibroblasts and smooth muscle cells (13, 14). Additionally, TGF-βs can induce or inhibit programmed cell death in different cell types, for example, apoptosis induced by TGF-βs plays a significant role in limb formation during development (15). In particular, TGF-βs are essential immunomodulatory molecular switches that play a bipolar role for different cell types, different cell states, or in concert with different cytokines and are therefore important for the homeostatic maintenance of the immune system (16). For example, inflammatory responses in their initial stages can be induced and enhanced by TGF-βs, and subsequently, inflammation is suppressed by TGF-βs, thus contributing to the regression of inflammation. However, the imbalance between the pro-inflammatory and immunosuppressive activities of TGF-βs leads to chronic inflammation and tissue fibrosis, as has been demonstrated in mouse models. For example, TGF-β1 knockout mice exhibit autoimmune inflammatory diseases characterized by inflammatory cell infiltration in multiple organs and the presence of autoantibodies in the circulatory system (17, 18), whereas TGF-β1 overexpressing mice exhibit tissue fibrosis and renal failure (19).

Although the function of TGF-βs in higher vertebrates has been exhaustively studied, the evolution of the function of TGF-βs is still unclear. Previous studies on TGF-βs in lower animals have focused on their functions involved in embryonic development, while when TGF-βs acquired immunomodulatory functions during evolution is a question that deserves in-depth investigation. Based on the conserved protein sequences of TGF-βs among different deuterostomes, previous studies have used cross-phyla experiments to provide clues that TGF-βs may perform similar functions in lower animals as in higher vertebrates. For example, amphioxus TGF-β could regulate the migration of mouse macrophage RAW 264.7 *in vitro* (20), while bovine TGF-β1 could affect the respiratory burst activity of rainbow trout macrophages (21). Additionally, TGF-βs have immunomodulatory functions similar to those of mammals in representative species of lower jawed vertebrates. For example, in the South African clawed frog (*Xenopus laevis*) TGF-β1 can suppress the proliferation of T cells (22). While goldfish (*Carassius auratus L.*) TGF-β1could induce the proliferation of goldfish fibroblast CCL71 and downregulate the TNF-α-activated nitric oxide response in macrophages (23). The Grass carp (*Ctenopharyngodon idellus*) TGF-β1 exerts a bidirectional regulatory effect on the proliferation of quiescent and activated peripheral blood leukocytes (24). Red seabream (*Pagrus major*) TGF-β can also regulate the migration of head kidney cells and peripheral blood leukocytes in a context-dependent manner (25). However, there is still a lack of sufficient data to support that TGF-βs are the most ancestral immunomodulatory cytokine in vertebrates.

Lamprey, the ancient species with a history of more than 350 million years, is one of the extant representatives of jawless vertebrates. As a direct ancestor of vertebrates, the lamprey provides a rich source of genetic information for the origin and evolution of vertebrates. Recently, the discovery of the adaptive immune system based on variable lymphocyte receptors (VLRs) in lampreys has overturned the traditional view that adaptive immunity exists only in jawed vertebrates and provided a key clue to the evolution of the adaptive immune system. Antibody-like molecules in the lamprey consist of three types of variable lymphocyte receptors (VLRs), which are membrane-bound proteins responsible for antigen recognition based on their highly diverse leucine-rich repeat (LRR) sequences (26–28). Among them, VLRAs and VLRCs are expressed by two different lymphocyte-like cells lineages similar to αβ and γδ TCR bearing T cells of jawed vertebrates, respectively. Additionally, VLRBs are expressed by B-like lymphocytes (29, 30). Although jawless vertebrates such as the lampreys employ unique antigen recognition receptors, the nature of the design principles and mechanisms of their immune system remains similar to those of jawed vertebrates (31). A complex intercellular communication system is known to coordinate the immune response process in jawed vertebrates, in which different lineages of immune cells can interact by releasing, sensing, and responding to a wide variety of cytokines. Previous studies on the gene expression patterns of several cytokines and their receptors (e.g., IL-8/IL-8R or IL-17/IL-17R) homologs suggest a possible functional interaction between T-like and B-like cells in lampreys (29, 32). However, there is still a lack of sufficient studies to elucidate how cytokines in lampreys mediate intercommunica tion between different immune effector cell lineages, which is valuable for understanding how the unique immune system in lampreys works.

In this study, we identified homologous gene members of the TGF-β subfamily in lampreys at the genome-wide level and analyzed the molecular evolutionary features of the family at the gene and protein levels using bioinformatics tools. We also investigated the changes in the expression levels of L-TGF-βs under quiescent and immuno-activated conditions, and analyzed the effects of recombinant L-TGF-β2 on the proliferation and apoptosis of lamprey immune cells. These results can help to further elucidate the evolution of the TGF-β subfamily in deuterostomes and reveal that TGF-βs may serve as one of the most ancestral immune regulators in vertebrates, with a significant role in the coordination between immune cells of lampreys.

## Materials and Methods

### Animals Maintenance and Cell Culture

Healthy adult Japanese lampreys (*Lampetra japonica*) with 30~50 cm in length, including males and females, were kept in fiber-reinforced plastic (FRP) tanks with a recirculating system at 10°C. For immune stimulation, the intraperitoneal injection was performed using 100 µg LPS (lipopolysaccharides; Sigma-Aldrich) or physiological saline solution. Then, animals were anesthetized in 100 mg/L MS222 (Aladdin) at 12 and 24 h post-immunization.

SMB cells and peripheral blood leukocytes were isolated as described previously (33). Briefly, the supraneural myeloid body tissues were stripped from lampreys followed by digestion by 0.1% type 1 collagenase (Sigma-Aldrich) at 4°C overnight. The obtained cell suspensions were further purified by Ficoll-Histophaque density gradient centrifugation (Sigma-Aldrich) at 400 × g for 30 min. After density gradient centrifugation was repeated once, purified SMB cells, which were retained at the interface of the density gradient, were washed with PBS twice. For isolation of leukocytes, briefly, blood from lampreys was collected through the cardiac puncture, and leukocytes were separated by Ficoll-Histophaque gradient centrifugation at 400 × g for 30 min. After counting under a microscope using trypan blue staining and hematocrit plates, cells were maintained in Leibovitz L-15 medium (Meilunbio) supplemented with 10% fetal bovine serum (HyClone), antibiotics (100 IU/ml of penicillin sulfate and 100 μg/ml of streptomycin) and 2 mM L-glutamine (Meilunbio). Cells (1~2×10^6^ cells/ml) were cultured in a low-temperature incubator at 18°C for the following studies.

### Identification of Candidate TGF-β Genes in Lampreys, Multiple Sequence Alignments, and Phylogenetic Analysis

The amino acid sequences of human TGF-β1~3 were used as baits, candidate members of the TGF-β subfamily in lampreys were screened in the genomic local databases of the reissner lamprey (*Lethenteron reissneri*), the genomic online databases of sea lamprey (*Petromyzon marinus*) (www.ncbi.nlm.nih.gov/genome/annotation_euk/Petromyzon_marinus/100/) and in the transcriptomic local database of the Japanese lamprey (*Lampetra japonica*) by the BLAST homology searching. Next, the conserved domains of each candidate TGF-β protein were verified using SMART and Pfam databases. Subsequently, the protein-coding sequence of each TGF-β gene and its chromosomal location (if available) were extracted. Sequences of members of the TGF-β subfamily in various representative species of deuterostomes were downloaded from the NCBI database (accession numbers are listed in **Supplementary Table 1**). Multiple sequence alignments were performed using ClustalW online tool (www.ebi.ac.uk/Tools/msa/), subsequently visualized and annotated by Jalview software (Version: 2.11.1.4) (34). Next, a phylogenetic tree of TGF-βs was constructed using the Maximum Likelihood method and JTT matrix-based model in MEGA X software (35). The tree with the highest log likelihood (-20554.46) is shown. Analysis of conserved motifs was performed online using MEME version 5.2.0 (http://meme-suite.org/tools/meme) with the following parameters: zero repetitions per sequence or one repetition per sequence, motif size between 8 and 50 aa, and a maximum of 20 motifs. The results were then visualized and annotated by TBtools software (Version: 1.075) (36).

### Structure Prediction of Genes and Proteins, and the Syntenic Relationship Analyses of TGF-β Genes

The TGF-β genes structure analyses were performed by the Ensembl database and TBtools software. The molecular model of lamprey TGF-β2 mature peptide homodimer was built by homology modeling of SWISS-MODEL (http://swissmodel.expasy.org/interactive) based on the human TGF-β2 (PDB code 2tgi.1.B) (37). Genomic synteny was obtained from Genomicus genome browser (version 104.02) (http://www.genomicus.biologie.ens.fr), Ensembl (http://asia.ensembl.org/index.html), and genomic databases of the reissner lamprey. Human TGF-β2 and TGF-β3 were used as reference genes, respectively. In cases of uncertain identity (e.g., a gene annotated with accession number, but without a definitive name), similarity searches in the Ensembl database were performed to establish possible homology between genes.

### Gene Cloning and Real-Time Quantitative PCR

Total RNA isolated from the livers of Japanese lampreys using MiniBEST universal RNA extraction kit (TaKaRa) was used to synthesize first-strand cDNA with PrimeScript™ II 1st strand cDNA synthesis kit (TaKaRa). The ORF of L-TGF-β2 and L-TGF-β3 cDNA was cloned using PrimeSTAR^®^ HS DNA polymerase. The PCR products were purified using the agarose purification kit (Meilunbio) and were cloned into the pEASY^®^-Blunt cloning vector (Transgen). Next, *E. coli* DH5α was transformed with the recombinant vector. Selected positive clones were sequenced by Sangon Biotech (Shanghai). To determine the transcriptional expression profiles of L-TGF-β2 and L-TGF-β3 of Japanese lamprey under normal physiological or immuno-activated conditions stimulating with a PAMP (lipopolysaccharide), relative gene expression in various tissues including gill, liver, intestine, kidney, supraneural myeloid body, heart, oral gland, and leukocytes were detected by real-time quantitative PCR assays. Briefly, total RNA was isolated from each tissue sample preserved in Biosample stabilizing reagent (Accurate Biology). Reverse transcription was then performed using PrimeScript™ RT reagent Kit with gDNA Eraser (TaKaRa). Controls without reverse transcriptase (No-RT) were prepared for each sample. qPCR was conducted with TB Green^®^ Premix Ex Taq™ II Kit according to the manufacturer’s protocol with lamprey Gapdh (GenBank accession No.KU041137.1) as an internal control. The heart cDNA template was diluted in a 1:5 ratio, and the C_t_ values of L-TGF-β2, L-TGF-β3, and Gapdh genes at each concentration gradient were measured by qPCR (7500 Real-Time PCR System, Applied Biosystems), the standard curves of the expression of these genes were finally plotted. Before qPCR assay, all primers were confirmed to have R^2^ greater than 0.98 in their standard curves, and all amplification efficiencies ranged from 90% to 105%. Furthermore, the specificity of the amplification reaction was analyzed by melt curve analyses. The primer sequences for gene cloning and qPCR are shown in **Supplementary Table 4**.

### Enzyme-Linked Immunosorbent Assay (ELISA)

Based on the expression of recombinant L-TGF-β2 protein and the screening of L-TGF-β antibody pairs in lampreys, a quantitative sandwich ELISA kit for the determination of relative mass values for natural L-TGF-β2/3 was developed. The standard curve was obtained using the four-parameter logistic (4-PL) curve-fit method with recombinant L-TGF-β2 protein as standards at concentrations between 20 and 640 pg/mL and 1:2 serial dilutions. SMB cells were stimulated with 10 μg/mL LPS, and PBS was added as a control, and both were incubated at 18°C for 12 h and 24 h. The medium supernatant was removed by centrifugation. To activate latent L-TGF-βs to the immunoreactive form, 1 N HCL was added to the supernatant for acidification, followed by 1 N NaOH for neutralization. Activated samples were added to the microplate and incubated for 2 h. After repeated washing, biotinylated antibodies were added and incubated for 1 h. Subsequently, horseradish peroxidase-labeled streptavidin was added and incubated for 30 min, followed by the addition of the substrate solution. The optical density at 450 nm was measured using a microplate reader (SpectraMax^®^ i3x, Molecular Devices) and corrected with the OD value at 570 nm. Experiments were repeated three times independently.

### Expression, Purification, and Refolding of Recombinant Proteins

Based on the sequence of the mature peptide in the L-TGF-β2 gene cloned above, artificial gene synthesis was performed after codon optimization to construct the recombinant plasmid pET28a-L-TGF-β2, which was transformed into *E. coli* Rosetta (DE3). Transformant was cultured overnight in LB broth containing 30 μg/mL kanamycin and 34 μg/mL chloramphenicol at 37°C. The cultures were diluted 1:100 with LB broth and subjected to further incubation until the OD value at 600 nm reached about 0.6. The expression of recombinant proteins was induced by adding isopropyl b-D-thiogalactoside (IPTG) to the cultures at a final concentration of 0.5 mM for 12 h at 20°C. Bacteria were sonicated and the inclusion bodies were resuspended in buffer C consisting of 50 mM Tris-HCL, 300 mM NaCl, 8 M urea, 0.1% Triton X-100 and 0.2% Triton X-114 (pH 8.0). The recombinant proteins were purified by chromatography on a Ni-NTA resin column. The purified proteins were refolded in renaturation buffer consisting of 20 mM Tris, 250 mM NaCl, 0.2 mM GSSG, 2 mM GSH (pH8.4) as described previously (38, 39), then dialyzed to storage buffer (20 mM Tris, 500 mM NaCl, 10% Glycerol, pH 8.4). The purified proteins were analyzed on a 12% non-reducing SDS-PAGE followed by Kaumas Brilliant Blue Staining. Further validation was performed using immunoblotting, using mouse anti-His-tag antibody (Sangon Biotech) as the primary antibody. Protein concentrations were determined by a non-interference protein assay kit (Sangon Biotech). Endotoxin levels of purified proteins were confirmed to be less than 1 EU/µg using an endotoxin detection kit (Xiamen Bioendo Technology).

### Cell Counting Kit-8 Assay and the BrdU Incorporation Assay

The effects of rL-TGF-β2 on the proliferation of MCF-7 cells and RAW 264.7 cells were analyzed using Cell Counting Kit-8 assay (CCK-8/WST-8). Briefly, MCF-7 cells and RAW 264.7 cells were maintained in DMEM medium (Meilunbio) supplemented with 10% fetal bovine serum (HyClone), antibiotics (100 IU/ml of penicillin sulfate and 100 μg/ml of streptomycin), 2 mM L-glutamine, and 30 mM HEPES (Meilunbio) in a CO_2_ incubator at 37°C. MCF-7 or RAW 264.7 cells suspensions were used for making 1:2 serial dilutions to each well of a 96-well plate. After adding CCK-8 reagent (Meilunbio) to each well and reacting for 4 h, standard curves were established by plotting the number of cells on the x-axis and the OD (450 nm) on the y-axis and then R^2^ greater than 0.98 was determined (data not shown). The cells were resuspended in DMEM medium containing 0.1% FBS, to which the indicated concentrations of L-TGF-β2 recombinant proteins were added, and incubated for 24 h, 48 h, and 72 h. The commercially available human TGF-β2 recombinant proteins (R&D Systems) were used as positive controls. At 4 h before the end of each incubation period, CCK-8 reagent was added to each well. Finally, the OD (450 nm) was measured using a microplate reader (SpectraMax^®^ i3x, Molecular Devices) and corrected with the OD (600 nm). The proliferation inhibition rate = [(Ac - As)/(Ac - Ab)] ×100%. As: absorbance of experimental wells in the presence of rL-TGF-β2. Ac: absorbance of control wells in the absence of rL-TGF-β2. Ab: absorbance of blank wells containing medium only.

The effects of rL-TGF-β2 on the proliferation of primary cultured SMB cells and leukocytes were analyzed using the Cell Proliferation ELISA BrdU colorimetric assay (Roche). Briefly, SMB cells and leukocytes were isolated and maintained as mentioned above. Cells were seeded in 96-well culture plates at a density of 1 × 10^5^ cells per well containing 100 µL L-15 medium/0.1% FBS with or without 10 μg/mL LPS and then treated with 10 μg/mL rL-TGF-β2, and incubated for 24 h, 48 h, 72 h, 96 h, and 120 h in a cell incubator at 18°C. At 24 h before the evaluation of mitogenesis using the BrdU ELISA, BrdU labeling reagent at a concentration of 10 mM was added to each well. The reaction was developed according to the manufacturer’s protocol. The OD (370 nm) was determined using a microplate reader (SpectraMax^®^ i3x, Molecular Devices) and corrected with the OD (492 nm). The stimulation index (SI) values = As/Ac. As: absorbance of experimental wells in the presence of rL-TGF-β2. Ac: absorbance of control wells in the absence of rL-TGF-β2. In control experiments, the colorimetric reaction was found to be directly proportional to the number of proliferating SMB cells and leukocytes in culture.

### Caspase-3/7 Activity Assay

To investigate the effect of rL-TGF-β2 on the apoptosis of primary cultured SMB cells and leukocytes induced by serum starvation, caspase-3/7 activity was analyzed using CellEvent™ Caspase-3/7 Green Detection Reagent (Thermo Fisher Scientific) according to the manufacturer’s protocol. Briefly, SMB cells and leukocytes were isolated and maintained as mentioned above. Cells were seeded in the black 96-well culture plates at a density of 1 × 10^5^ cells per well containing 100 µL L-15 medium without FBS. Then cells were treated with 0.01 μg/mL or 1 μg/mL rL-TGF-β2, respectively, and incubated for 6 h and 30 h in a cell incubator at 18°C. The caspase-3/7 inhibitor Ac-DEVD-CHO was used at 20 μM to block apoptosis. At 30 min before the evaluation of caspase-3/7 activity, caspase-3/7 Green Detection Reagent at a concentration of 5 µM was added to each well. Fluorescence density (Excitation wavelength 485 nm, emission wavelength 515 nm) was determined using a microplate reader (SpectraMax^®^ i3x, Molecular Devices). The same cell density between each well was subsequently confirmed by cell counting. In control experiments, the fluorescence intensity was found to be proportional to the number of SMB cells induced to apoptosis by serum deprivation.

### Statistical Analysis

All data represent the mean ± SEM of at least three independent experiments (n) in which each condition was tested at least in duplicate. Two-tailed unpaired *t*-test, multiple *t*-tests, two-way RM ANOVA with *post-hoc* Dunnett’s multiple comparison test and ordinary one-way ANOVA with *post-hoc* Tukey’s multiple comparison test was performed where appropriate using GraphPad Prism 8. *: *p* < 0.05, **: *p* < 0.01.

## Results

### Identification and Evolutionary Analyses of TGF-β Subfamily in the Lamprey Genomes

In this study, to investigate the origin and evolution of the TGF-β subfamily in vertebrates, genome-wide identification of the TGF-β homologs of the lamprey was performed using the amino acid sequences of human TGF-β1~3 for homology alignment in the genomic databases of the reissner lamprey (*Lethenteron reissneri*) and the sea lamprey (*Petromyzon marinus*) and in the transcriptomic database of the Japanese lamprey (*Lampetra japonica*). As a result, eight candidate sequences containing conserved prodomains and ligand domains were identified in three species of lampreys. Based on phylogenetic analysis and sequence homology comparison, we further found that these sequences represented two classes of TGF-β subfamily members. Among them, the TGF-β2 gene had two homologous gene copies, TGF-β2A and TGF-β2B, in both reissner lamprey and sea lamprey, respectively, and only one TGF-β2 homologous gene was found in Japanese lamprey. Furthermore, a TGF-β3 homologous gene was present in all three species of lampreys. However, no homolog of the TGF-β1 gene was found in any of the three lampreys.

**Figure 1:**
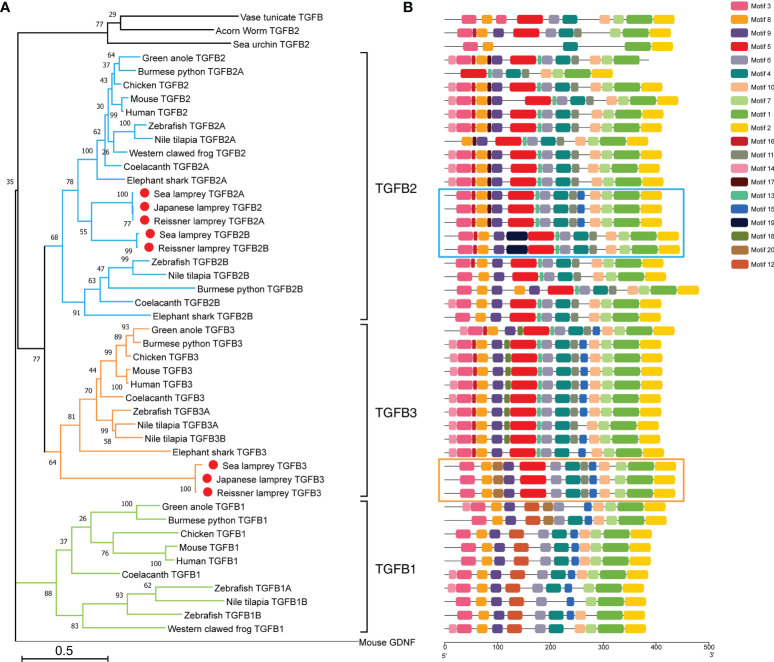
Phylogenetic analysis and conserved motif compositions of TGF-βs in the lamprey and other representative species of the deuterostomes. **(A)** Phylogenetic tree of TGF-β subfamily members based on the Maximum Likelihood method and JTT matrix-based model. This ML tree consists of 47 full-length amino acid sequences of TGF-βs identified in lampreys (highlighted with red dots) as well as representative species of the deuterostomes. The tree is drawn to scale, where the bar representing genetic distance is 0.5, and the tree is rooted with the GNDF sequence of mouse (*Mus musculus*). Bootstrap supports in interior branches are shown. The full names of species and the corresponding accession numbers of TGF-β proteins are listed in **Supplementary Table 1**. **(B)** Conserved motifs of TGF-β subfamily members. The motif compositions of all TGF-βs included in the phylogenetic tree are mapped in equal proportion to the number of amino acid residues. Among them, the motifs of five TGF-β2 homologs in lampreys are highlighted in blue boxes, and the motifs of three TGF-β3 homologs in lampreys are highlighted in orange boxes. The conserved motifs numbered from 1 to 20 were indicated by different colored boxes, and the matching amino acid sequences in motifs are listed in **Supplementary Table 2**.

To investigate in detail the evolutionary position of the TGF-βs in lampreys, we constructed a phylogenetic tree including the full-length sequences of TGF-βs identified in lampreys and the TGF-β sequences of representative species in the deuterostomes using the Maximum Likelihood method (**Figure 1A**). Consistent with previous studies (40), we used mouse GDNF (glial-derived neurotrophic factor) as an outgroup to root the phylogenetic tree. GDNF shares key structural similarities with mature TGF-β family ligands, such as having the same cysteine pattern as TGF-β family ligands and belonging to dimeric secretory proteins, but signal through a Ret tyrosine kinase receptor that is distinct from the TGF-β family receptors. Therefore, GDNF is not recognized as a member of the TGF-β family. As shown in **Figure 1A**, the overall topology of the phylogenetic tree of the TGF-β subfamily consisted of two major clusters, with TGF-β1 forming one phylogenetic cluster and TGF-β2, TGF-β3, and the lamprey TGF-β homologs in the other. In the latter cluster, the lower deuterostomes, including acorn worm in hemichordates, sea urchin in echinoderms, and sea squirts in tunicates (urochordates), all had only one homologous sequence of TGF-β and were located at the bottom of the cluster. Subsequently, TGF-β2 and TGF-β3 clustered into one cluster and diverged, respectively. Among them, the cluster of TGF-β2 further diverged into two branches, TGF-β2A and TGF-β2B. The cluster of TGF-β2B included only a few species including cartilaginous and bony fish, which may be the result of genome-wide replication in fish. In contrast, the cluster of TGF-β2A contained jawed vertebrates including cartilaginous fish, bony fish, amphibians, reptiles, birds, mammals, and jawless species represented by the lamprey, of which the branch consisting of five homologous sequences of the lamprey was located at the bottom of the TGF-β2A cluster. The topology of the phylogenetic tree showed that there was little difference between the lamprey TGF-β2A and TGF-β2B, and they may both represent ancestral genes of vertebrate TGF-β2A, but lamprey TGF-β2B was relatively more distantly related to TGF-β2B in fish. Furthermore, the phylogenetic cluster of TGF-β3 included all species of jawless and jawed vertebrates, with the three TGF-β3 homologs of lampreys located at the bottom of the cluster, suggesting its primitive position in the molecular evolution of vertebrate TGF-β3.

Protein domain analysis showed that all lamprey TGF-β family members had the same domains as human TGF-βs, including the N-terminal prodomain as well as the C-terminal ligand domain, and all contained an N-terminal signal peptide. We further analyzed and compared the motifs of all TGF-β homologs included in the phylogenetic tree (**Figure 1B**). Among the 20 predicted conserved motifs, TGF-β2A in the reissner lamprey and the sea lamprey, as well as TGF-β2 in the Japanese lamprey, contained the same 15 motifs (motif 1, 2, 3, 4, 5, 6, 7, 8, 9, 10, 11, 13, 15, 16, 17). Among them, motif 3, 4, 5, 6, 8, 9, 10, 11, 13, 15, 16, 17 formed the prodomain of TGF-β, while motif 1, 2, 7 formed the ligand domain. Except for motif 15, which was present only in TGF-β1 and 2, the remaining 14 motifs were conserved in jawed vertebrates including cartilaginous fish, bony fish, and mammals. Additionally, motifs 13, 15, and 17 started to appear in lamprey TGF-βs compared to TGF-β homologs in lower deuterostomes, including acorn worm, sea urchin, and sea squirt. Notably, TGF-β2B, another gene copy of TGF-β2 in both the reissner lamprey and sea lamprey, contained 14 motifs (motif 1, 2, 3, 4, 5, 6, 7, 8, 9, 10, 11, 13, 16, 19). Compared with the lamprey TGF-β2A, TGF-β2B lacked motifs 15 and 17 but had motif 19 that was present only in lampreys. For TGF-β3, all three lamprey TGF-β3 sequences contained the same 13 motifs (motif 1, 2, 3, 4, 5, 6, 7, 8, 9, 10, 11, 15, 20). Except for motif 20, the remaining 12 motifs were conserved in higher vertebrates. Thus, the comparison of motifs in the TGF-β homologs of lampreys and other species showed that motifs constituting the highly conserved TGF-β ligand domains were almost identical, and the differences were mainly in the types of motifs constituting the prodomains. Moreover, the majority of motifs of each member of the TGF-β family in lampreys have been preserved in higher animals and contained some motifs that were absent in lower species, further indicating the importance of the lamprey TGF-βs in the evolution of the TGF-β subfamily of vertebrates.

**Figure 2:**
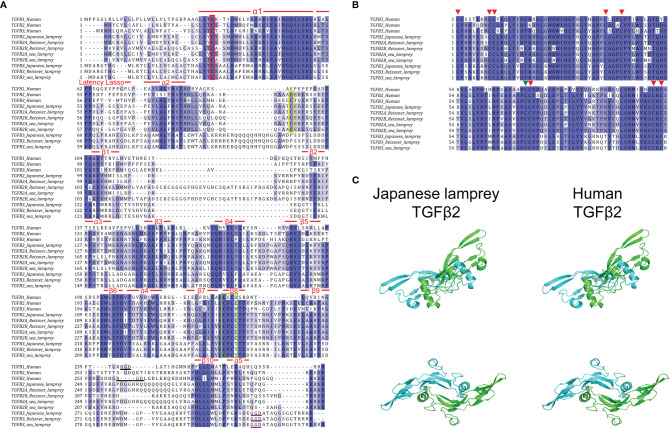
Amino acid sequence alignment of TGF-βs between lampreys and humans. **(A)** Alignment of the prodomains of TGF-βs between lampreys and humans. The sequence features of the straitjacket domain and the arm domain are shown above the alignment. Conserved cysteine residues involved in the formation of the TGF-β large latent complex (LLC) are highlighted in the red box, green box, and green box, respectively. The Arg-Gly-Asp (RGD) sequences in human TGF-β1 and TGF-β3 are highlighted by black underline. The Gly-Gly-Asp (GGD) sequences in lamprey TGF-β3 are highlighted by purple underline. **(B)** Alignment of the ligand domains of TGF-βs between lampreys and humans. The eight conserved cysteine residues forming four intramolecular disulfide bonds are marked by the red arrows above the alignment. The other cysteine residue involved in the formation of intermolecular disulfide bonds that link the two monomers into a dimer is marked by the green arrow. **(C)** Protein structure comparison of TGF-β2 mature peptide between Japanese lamprey and human. The molecular model of lamprey TGF-β2 mature peptide homodimer was built by homology modeling of SWISS-MODEL based on the human TGF-β2 (PDB code 2tgi.1.B). The two monomers of TGF-β2 are shown in blue and green, respectively. Two views are displayed, rotated by 90° with respect to each other.

As previously reported, the full-length sequences of TGF-βs from jawed vertebrates can be cleaved into propeptides and ligands based on the furin cleavage site RX [R/K] R (41, 42). Consistent with members of the human TGF-β subfamily, lamprey TGF-βs had nearly identical RKKR cleavage sites, except for the RRRR of the sea lamprey TGF-β3 (**Figure 2A**, the last four residues in each sequence). Additionally, the sequence features of the prodomain previously found in mammalian TGF-β1 (43) were significantly conserved in each member of the lamprey TGF-β subfamily compared to humans (**Figure 2A**). Four features (α1, Latency Lasso, α2, and β1) of the straitjacket domain and seven features (helices β2~β6 and α4) of the arm domain in the lamprey TGF-β had multiple highly conserved amino acid residues. Notably, the human TGF-β1 prodomain contained three cysteines, one of which formed a disulfide bond with LTBP, and the other two formed intermolecular dimerization links in the “bowtie” region of the LAP structure. These cysteines were important for the formation of the TGF-β large latent complex (LLC) and therefore the pattern of cysteine residues in the prodomain was relatively conserved across multiple species (43). Our study demonstrated that the pattern of cysteine residues in the prodomain of the lamprey was conserved but moderately different compared to that of humans. First, there was a cysteine in α1 of all lamprey TGF-β homologs, which was identical to C33 in human TGF-β (**Figure 2A**, red box), suggesting that the LAP formed by the lamprey TGF-β prodomain was also covalently linked to LTBP *via* a cysteine bridge (44, 45). Secondly, the “bowtie” region of β8 in all lamprey TGF-β homologs also had two adjacent cysteines forming the C-X-C motif, which was identical to that of human TGF-βs, especially the C-P-C-C motif in the lamprey TGF-β2 and human TGF-β2 (Fig2A, green box), suggesting that lamprey LAP can also form homodimers through disulfide bonds. Additionally, both human TGF-β2 and TGF-β3 LAPs contained more cysteine residues (C89/C91) that contributed to the formation of additional crosslinks between the LAP monomers. However, these cysteine residues were conserved only in lamprey TGF-β2 and were not found in TGF-β3 (**Figure 2A**, yellow box). Also, another conserved cysteine residue (C123) in human TGF-β3 was not found in lamprey TGF-β3. Our findings suggested that these additional disulfide bonds may not be necessary for the structural stability of the LAP dimer.

In higher vertebrates, the cell surface integrins αvβ6 and αvβ8 bind to the Arg-Gly-Asp (RGD) sequences present in the propeptides of TGF-β1 and TGF-β3 (**Figure 2A**, black underline) and activated the latent complex of TGF-β through this interaction, whereas the RGD sequence was absent in TGF-β2 (46–48). In contrast to the strict conservation of the RGD motif in the prodomain of the TGF-β3 previously found in most deuterostomes, we did not find an RGD sequence in lamprey TGF-β3, but only a Gly-Gly-Asp (GGD) sequence near the C-terminus of the prodomain (**Figure 2A**, purple underline). This finding indicated that the lamprey TGF-β3 may be activated by alternative mechanisms that have not been elucidated. Given that lower deuterostomes, including sea urchins, tunicates, and hemichordates, had RGD motifs in their TGF-β prodomain, it is suggested that the lack of Arg in the RGD motif in lamprey TGF-β3 was an acquired rather than an ancestral feature.

Consistent with previous studies (49), we defined the ligand on the first conserved cysteine of the TGF-β mature peptide (ligand domain). The results of multiple sequence alignment between the ligand domains of lamprey TGF-β2~3 and human TGF-β1~3 identified significant amino acid conservation of TGF-β ligands across species (**Figure 2B**). In all pairwise sequence alignments, the amino acid identity of the TGF-β ligand domain was more than 70%. For example, the Japanese lamprey TGF-β2 ligand showed 84% identity compared to human TGF-β2, 81% identity compared to human TGF-β3, and 75% identity compared to human TGF-β1. Additionally, the Japanese lamprey TGF-β3 ligand showed 75% identity compared to human TGF-β3, 73% identity compared to human TGF-β2, and 70% identity compared to human TGF-β1. Importantly, the lamprey TGF-βs ligand domains showed nine cysteine residues at the same position compared to humans, with eight cysteines forming four intramolecular disulfide bonds (**Figure 2B**, red arrows) and the other cysteine responsible for the formation of intermolecular disulfide bonds that link the two monomers into a dimer (**Figure 2B**, green arrow). This cysteine pattern was conserved not only in the true TGF-β subfamily but also in the TGF-β superfamily members of many species. The characteristic cysteine pattern resulted in the TGF-β proteins exhibiting a three-dimensional structure with a cysteine knot motif. The protein structure prediction revealed that the Japanese lamprey TGF-β2 mature peptide displayed the same secondary structure as that of humans with a largely conserved α-helix and nine β-sheets. And the lamprey TGF-β2 mature peptide had the same dimeric structure as the human homolog, where the central 3-1/2 turn helix of one monomer rested on the concave surface formed by the β chain of the other monomer (**Figure 2C**). Therefore, the evolutionary conservation of the TGF-β ligand domains in the lampreys suggested that they may have receptor-binding activity and biological functions remarkably similar to those of higher vertebrates.

**Figure 3:**
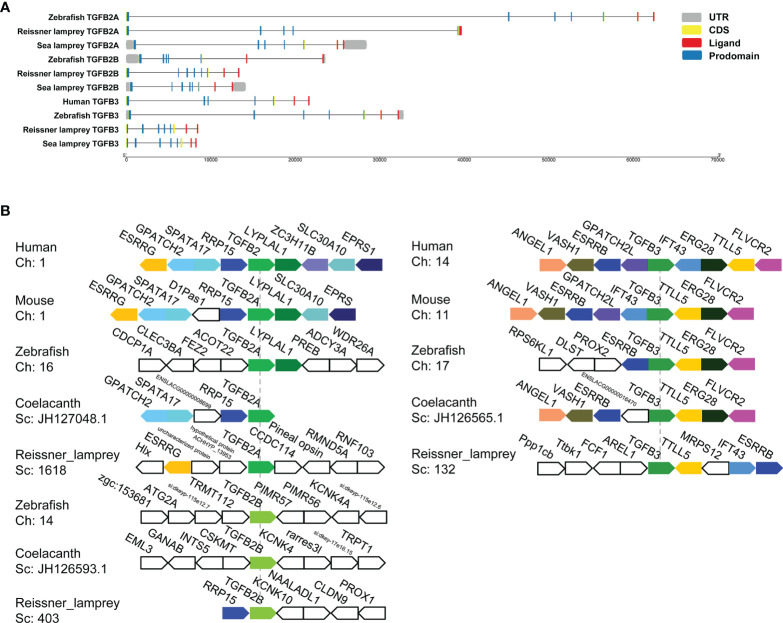
Gene structures and syntenic relationships of TGF-β2A/2B and TGF-β3 in the reissner lamprey, bony fishes, and mammals. **(A)** The structure of TGF-β2A/2B and TGF-β3 genes of different species. Gene lengths are plotted according to the scale at the bottom. The 5’ and 3’ untranslated regions are represented by grey boxes. Exons are represented by yellow boxes, and introns are represented by straight lines. In each protein-coding sequence, the part encoding prodomain is further marked in blue, while the part encoding ligand is marked in red. **(B)** The syntenic relationship analyses for comparing the neighboring gene environments of TGF-β2A/2B and TGF-β3 in different species. Human TGF-β2 and TGF-β3 were used as reference genes, respectively, and the adjacent genes of TGF-β conserved in humans and other species were labeled with different colored polygons. Ch: Chromosome No., Sc: Scaffold No., opposite polygons represent genes on opposite strands. The accession numbers of TGF-β genes in different species retrieved from Genomicus are listed in **Supplementary Table 2**.

To explore the evolutionary changes in the structure of the TGF-β genes, the gene structures of lampreys, zebrafish, and humans were mapped (**Figure 3A**). Compared to the zebrafish TGF-β2A gene, both the sea lamprey TGF-β2A and zebrafish contained seven exons, while the reissner lamprey TGF-β2A contained fewer exons. Second, compared with the zebrafish TGF-β2B gene, which contained seven exons, there was one more exon of the TGF-β2B gene in reissner lamprey and sea lamprey. Furthermore, the TGF-β3 gene had one more exon in reissner lamprey and sea lamprey compared to the human and zebrafish TGF-β3 genes, which contain seven exons. Additionally, the length of introns in the TGF-β gene of the lamprey was also generally shorter than in higher animals. Although the number of exons of TGF-βs differed slightly between lampreys and higher vertebrates, their ligand domains were all encoded by the last two exons, which further confirmed the highly conserved nature of the ligand domain of TGF-β during evolution.

For enhancing the phylogenetic-based analysis of the evolutionary history of the vertebrate TGF-β family, we performed the syntenic relationship analyses comparing the neighboring gene environments of TGF-βs between bony fishes (coelacanth and zebrafish), mammals (mice and humans), and the reissner lamprey (**Figure 3B**). First, the genes adjacent to TGF-β2A and TGF-β3 were relatively conserved in jawed vertebrates but differed significantly compared to the genes adjacent to the TGF-β2B gene in bony fishes. Importantly, the ESRRG gene could be detected in the proximity of TGF-β2A in lampreys, and there was also a shared syntenic relationship between the ESRRG gene and TGF-β2 in humans and mice. Additionally, the region where TGF-β2B was located in lampreys varied greatly compared to TGF-β2B in bony fishes, and there was no conserved linkage gene between them. The lamprey TGF-β2B was instead closely adjacent to the RRP15 gene, which was consistently present in the syntenic region of human and mouse TGF-β2 as well as coelacanth TGF-β2A. This finding suggested that the lamprey TGF-β2B was not a direct ancestor of bony fish TGF-β2B, but was more closely related to TGF-β2A in jawed vertebrates. Meanwhile, the syntenic relationship of lamprey TGF-β3 was relatively similar to that of jawed vertebrates. Although the order of gene arrangement varied across species, TGF-β3 was consistently linked to TTLL5 and ESRRB in humans, mice, zebrafish, coelacanth, and lampreys, IFT43 was also consistently present in the adjacent regions of human, mouse, and lamprey TGF-β3. Thus, the chromosomal regions where members of the TGF-β subfamily of the lamprey resided were relatively conserved in their adjacent gene environment during evolution compared to jawed vertebrates, despite undergoing somewhat chromosomal rearrangement.

### Expression Profiles of L-TGF-βs in Lampreys

**Figure 4:**
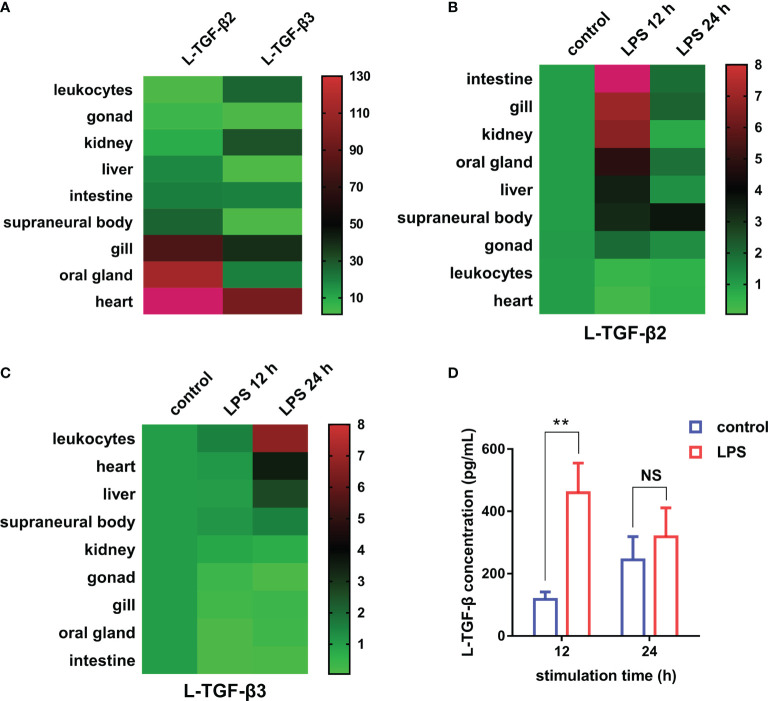
Expression profiles of TGF-βs in the Japanese lamprey. **(A)** Heatmap of the tissue distribution of L-TGF-β2 and L-TGF-β3 determined by real-time PCR. For L-TGF-β2, its relative expression level in leukocytes was set to 1, while for L-TGF-β3, its relative expression level in the liver was set to 1. Colorized matrixes highlight the high expressing tissues in red, the low expressing tissues in green and the median expression in black as shown in the color scale. **(B, C)** Heatmaps of the changes in L-TGF-βs expression levels before and after LPS immunization. The expression levels of L-TGF-β2 **(B)** and L-TGF-β3 **(C)** after 12 and 24 hours of LPS stimulation relative to those in unimmunized lampreys were analyzed using real-time PCR. The green and red in colorized matrixes indicate low to high expression levels, respectively. The experiments were repeated 3 times independently. **(D)** Changes in the level of L-TGF-β proteins secreted by SMB cells. The total amount of L-TGF-βs including the latent and activated forms in the supernatant of primary cultured SMB cells after 12 and 24 hours of *in vitro* LPS stimulation were determined using a customized sandwich ELISA kit. The standard curve is shown in **Supplementary Figure 1**. Error bars represent mean ± SEM from three independent experiments. Statistical significance was calculated using a two-tailed unpaired *t*-test. **: *p* < 0.01, NS, Not significant.

First, to examine the transcriptional expression profiles of L-TGF-β2 and L-TGF-β3 in various tissues of Japanese lamprey, we selected gill, liver, intestine, kidney, supraneural myeloid body, heart, oral gland, and leukocytes for real-time quantitative PCR assays. The results showed that tissue expression profiles of L-TGF-β2 and 3 were different at the transcriptional level (**Figure 4A**). The expression of L-TGF-β2 was lowest in leukocytes and highest in the heart, where it was about 400 fold higher than that in leukocytes. Additionally, L-TGF-β2 expression in oral glands and gill tissues was about 120~150 fold higher than that in leukocytes, whereas in supraneural body, kidney, liver, and intestine it was about 10~30 fold higher than that of leukocytes. Furthermore, the expression of L-TGF-β3 was lowest in the liver, while a predominant expression was also found in the heart. L-TGF-β3 expression in other tissues including the intestine, oral glands, leukocytes, kidney and gill is about 20~40 fold higher than that in the liver, except in supraneural body where the expression is similar to that in the liver. We further performed a single injection with lipopolysaccharide (LPS) to mimic bacterial infestation of Japanese lampreys, thereby activating the innate immune response of lampreys, and subsequently examined the altered transcript levels of L-TGF-βs in various tissues before and after immunization using real-time quantitative PCR (**Figure 4B**). The results showed that L-TGF-β2 was significantly upregulated in the intestinal, gill, kidney, oral gland, liver, supraneural body and gonads 12 hours after immune activation compared to controls, and conversely was significantly downregulated in leukocytes and heart. Alternatively, L-TGF-β3 was significantly upregulated in leukocytes, heart, liver, and supraneural body compared to control 24 h after immune activation, but significantly downregulated in the kidney, gonads, gills, oral glands, and intestine (**Figure 4C**). These results demonstrated that the TGF-β family may contribute to immune regulation in the lamprey. Moreover, L-TGF-β2 and L-TGF-β3 differed significantly in response patterns to lipopolysaccharide stimuli. After LPS immunization, L-TGF-β2 expression was rapidly upregulated in most tissues and then fell back to basal levels 24 hours after stimulation. In contrast, L-TGF-β3 expression was upregulated only in a limited number of tissues, and the upregulation of L-TGF-β3 was slower and more modest compared to the change in L-TGF-β2 expression. For example, the maximum upregulation of L-TGF-β2 expression in all tissues after stimulation occurred in the intestine, which was upregulated 23-fold at 12 h, but the maximum upregulation of L-TGF-β3 expression was in leukocytes, which was upregulated 7-fold at 24 h. These differences imply that L-TGF-β2 may play a central regulatory role in the innate immune response of the lamprey. To further verify the effect of LPS on the level of L-TGF-β proteins secreted by SMB cells (supraneural myeloid body cells), we examined the total amount of L-TGF-βs including the latent and activated forms in the supernatant of primary cultured SMB cells based on the standard curve (**Supplementary Figure 1**) using ELISA, and compared the total amount of L-TGF-β proteins after 12 and 24 hours of *in vitro* LPS stimulation of SMB cells, respectively. As shown in **Figure 4D**, L-TGF-βs protein levels were found to be significantly higher in the LPS-treated group than in the control after 12 h. These results revealed that LPS stimulation of SMB cells resulted in a significant increase in the total amount of L-TGF-βs secreted into the cell supernatant compared to the control, which is consistent with previous results from real-time quantitative PCR (**Figures 4B, C**).

### Expression and Purification of Recombinant L-TGF-β2 Mature Peptide

**Figure 5:**
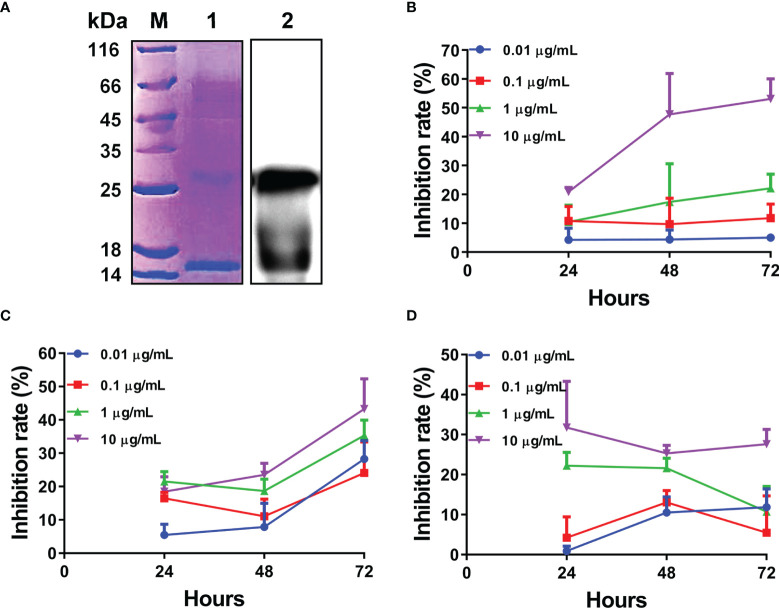
Expression and biological activity analysis of recombinant L-TGF-β2 mature peptide. **(A)** Detection of L-TGF-β2 recombinant protein using SDS-PAGE and immunoblotting under non-reducing conditions. The bands of rL-TGF-β2 monomer and dimer are found at about 13 kDa and 26 kDa, respectively. **(B)** The inhibition rate of rL-TGF-β2 on the proliferation of MCF-7 cells using CCK8 assay. Two-way RM ANOVA is used for statistical analysis. Time: F (2, 16) = 6.148, *P* = 0.0105; dose: F (3, 8) = 86.53, *P* < 0.0001; the interaction of time and dose: F (6, 16) = 2.886, *P* = 0.0421. **(C)** The inhibition rate of rL-TGF-β2 on the proliferation of RAW 264.7 cells using CCK8 assay. Two-way RM ANOVA is used for statistical analysis. Time: F (2, 24) = 148.4, *P* < 0.0001; dose: F (3, 12) = 7.894, *P* = 0.0036; the interaction of time and dose: F (6, 24) = 6.049, *P* = 0.0006. **(D)** The inhibition rate of rL-TGF-β2 on the proliferation of LPS-stimulated RAW 264.7 cells using CCK8 assay. Two-way RM ANOVA is used for statistical analysis. Time: F (2, 16) = 1.296, *P* = 0.3009; dose: F (3, 8) = 39.55, *P* < 0.0001; the interaction of time and dose: F (6, 16) = 2.753, *P* = 0.0493. All experiments were repeated thrice.

Given the rapid and significant upregulation of L-TGF-β2 expression following lipopolysaccharide stimulation, it is suggested that L-TGF-β2 may play an important role in the innate immune response of the lamprey. To further reveal its possible regulatory functions, we performed recombinant expression of the mature peptide fragment of the Japanese lamprey L-TGF-β2 using a prokaryotic expression system. To promote the formation of homodimers of recombinant L-TGF-β2 mature peptide through inter- and intra-chain disulfide bonds, we performed *in vitro* refolding of the fusion protein using the glutathione redox system consisting of GSH (reduced glutathione) and GSSG (oxidized glutathione). The refolded protein was subsequently analyzed by non-reducing SDS-PAGE (**Figure 5A**, left), and the bands of recombinant protein monomer and dimer were found at about 13 kDa and 26 kDa, respectively, which were nearly consistent with the predicted theoretical molecular weights. The results demonstrated that the L-TGF-β2 recombinant protein formed homodimers through disulfide bonds after protein renaturation. To confirm the purity of the L-TGF-β2 recombinant protein, we further performed immunoblotting analysis using an anti-His tag antibody. The results showed (**Figure 5A**, right) that the monomer and dimer of the recombinant protein appeared as specific bands at the corresponding molecular weight, respectively, indicating a good purity and correct size of the recombinant protein. We also performed mass spectrometry analysis of the purified products and found that the identified sequences matched with the predicted sequences of L-TGF-β2 (data not shown).

For verifying the biological activity of recombinant L-TGF-β2, we further analyzed the effect of rL-TGF-β2 protein on the proliferation of MCF-7 cells using the CCK8 assay. After the cells were treated with different doses of rL-TGF-β2 for 24, 48, and 72 h, the OD values were measured at 450 nm and the inhibition rate of rL-TGF-β2 on the proliferation of MCF-7 cells was determined. As shown in **Figure 5B**, it was found that the inhibition rate of MCF-7 cell proliferation reached 60% after 10 ug/ml rL-TGF-β2 stimulation for 72 h, while the inhibition rate was about 20% after 1 ug/ml rL-TGF-β2 incubation for 72 h and 0.1 ug/ml rL-TGF-β2 inhibition rate was about 10%. The statistical analysis illustrated that the proliferation inhibition rate displayed a significant positive correlation with rL-TGF-β2 treatment dose or treatment time, which was similar to the effect of human TGF-β2 on MCF-7 cell proliferation (**Supplementary Figure 2**). We next examined the effect of rL-TGF-β2 on the proliferation of the mouse macrophage RAW 264.7. The inhibition rate of rL-TGF-β2 on the proliferation of RAW 264.7 cells is shown in **Figure 5C**, and it was found that the inhibition rate reached 40% after 10 ug/ml rL-TGF-β2 stimulation for 72 h, while the inhibition rate was about 30% after 1 ug/ml rL-TGF-β2 incubation for 72 h and 0.1 ug/ml rL-TGF-β2 inhibition rate was about 20%. The statistical analysis also illustrated that RAW 264.7 cells, similar to MCF-7 cells, showed the same significant positive correlation of proliferation inhibition rate with rL-TGF-β2 treatment dose or treatment time. Additionally, we further analyzed the effect of different doses of rL-TGF-β2 on the proliferation of RAW 264.7 cells after lipopolysaccharide activation. As shown in **Figure 5D**, different doses of rL-TGF-β from 0.01 ug/ml to 10 ug/ml were effective in inhibiting the proliferation of RAW 264.7 cells after LPS activation. Statistical analysis showed that the proliferation inhibition rate of activated RAW 264.7 cells was significantly and positively correlated with the rL-TGF-β2 treatment dose, but not with the treatment time. Therefore, our results showed that L-TGF-β2 can effectively inhibit the proliferation of MCF-7 and RAW 264.7 cells, indicating that the lamprey TGF-β homolog was functionally conserved, and confirming that rL-TGF-β2 can be used for further functional studies because of its good biological activity.

### The Regulatory Effect of L-TGF-β2 on the Proliferation of SMB Cells and Leukocytes in the Lamprey

**Figure 6:**
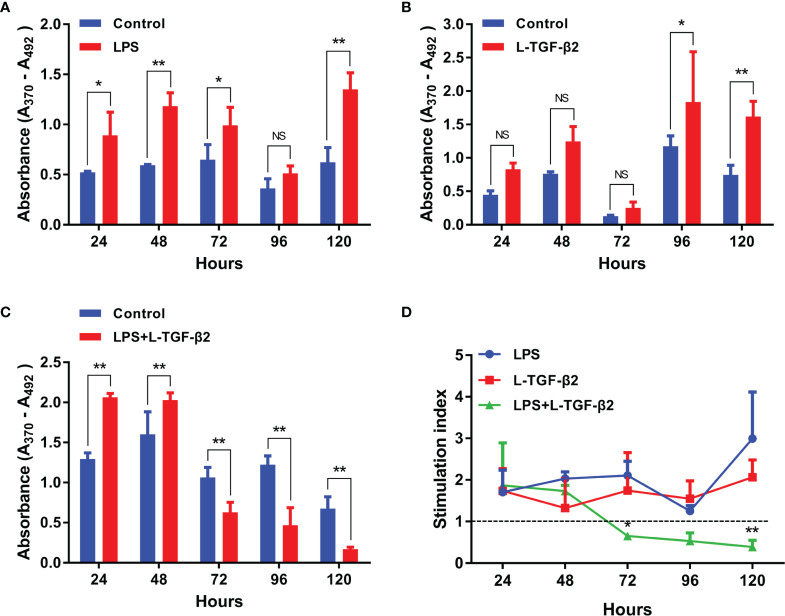
The effect of rL-TGF-β2 on the proliferation of quiescent and activated SMB cells in lampreys detected using the BrdU incorporation method. **(A)** The effect of LPS on the proliferation of SMB cells. The OD values are compared between the LPS-treated group with the control after 24, 48, 72, 96, and 120 h of incubation. **(B)** The effect of rL-TGF-β2 on the proliferation of quiescent SMB cells. The OD values are compared between the rL-TGF-β2-treated group with the control after 24 to 120 h of incubation. **(C)** The effect of rL-TGF-β2 on the proliferation of LPS-stimulated SMB cells. The OD values are compared between the rL-TGF-β2-treated group with the control after 24 to 120 h of incubation. In Figures **(A–C)**, the statistical analyses are all performed by multiple *t*-tests, in which the statistical significance is determined using the Holm-Sidak method, with alpha = 0.05. *: Adjusted *p* < 0.05, **: Adjusted *p* < 0.01, NS, Not significant. All experiments were repeated thrice. **(D)** Trends in the stimulation index of SMB cells proliferation under different treatment conditions. Two-way RM ANOVA is used for statistical analysis. Treatment condition: F (2, 6) = 17.56, *P* = 0.0031; Treatment time: F (4, 24) = 2.135, *P* = 0.1075; Interaction: F (8, 24) = 3.18, *P* = 0.0133. The LPS-treated group was subsequently set as the control for Dunnett’s multiple comparison test. LPS-treated group vs. LPS + rL-TGF-β2 co-treated group: *:*P* = 0.0072 (72 h), **:*P* < 0.0001 (120 h).

For investigating the effect of L-TGF-β2 on the proliferation of primary cultured SMB cells, we used ELISA-based detection of the incorporation of the thymidine analog BrdU as a method for the measurement of *in vitro* mitogenesis. Firstly (**Figure 6A**), when SMB cells were stimulated by LPS, the OD values were significantly higher in the LPS-treated group compared with the control after 24, 48, 72, and 120 h of incubation, indicating that LPS can induce the proliferation of SMB cells. Alternatively, when SMB cells were stimulated with rL-TGF-β2 (**Figure 6B**), the OD values of the treated group were significantly higher than the control after 96 h as well as 120 h, which indicated that L-TGF-β2 could promote the proliferation of quiescent SMB cells. Finally, the OD values of SMB cells were significantly higher in the treated group compared with the control after 24 and 48 h of co-stimulation with LPS and rL-TGF-β2. However, with the increase of incubation time, the OD values of the treated group were significantly lower than the control at 72, 96, and 120 h (**Figure 6C**). This result indicated that L-TGF-β2 could inhibit the proliferation of LPS-activated SMB cells. The stimulation index (SI) was further analyzed based on the OD values measured using the BrdU method to obtain the trend of SI values of SMB cells proliferation under different treatment conditions (**Figure 6D**). Firstly, the trend of SI changes under stimulation with rL-TGF-β2 was found to be similar to that under LPS stimulation, indicating that SMB cell proliferation was promoted under both conditions. However, the SI values gradually decreased during LPS and rL-TGF-β2 co-stimulation, where the SI was less than 1 after 72 h of incubation, indicating that SMB cell proliferation was inhibited. Statistical analysis also showed a significant difference in the trend of SI values in the LPS and rL-TGF-β2 co-stimulation group compared with the LPS stimulation group. These results revealed that L-TGF-β2 could promote the proliferation of SMB cells in the quiescent state and inhibit the proliferation of SMB cells in the activated state.

Additionally, we also investigated the effect of L-TGF-β2 on the proliferation of peripheral blood leukocytes in lampreys. It was found that leukocytes had significantly higher OD values compared with the control at 24 and 48 h post-LPS stimulation, indicating that LPS can induce the proliferation of leukocytes (**Supplementary Figure 3A**). Alternatively, when leukocytes were stimulated by rL-TGF-β2 (**Supplementary Figure 3B**), the OD values of the treated group were significantly lower than those of the control group after 24 and 48 h, indicating that L-TGF-β2 could inhibit the proliferation of quiescent leukocytes. When leukocytes were co-stimulated by LPS and rL-TGF-β2, the OD values of the treated group also decreased significantly after 24, 48, and 72 h of incubation compared with the control (**Supplementary Figure 3C**), which suggested that L-TGF-β2 could inhibit the proliferation of leukocytes after LPS activation. Similar to the analysis for SMB cell proliferation, the trends of SI values of leukocytes at various time points under different treatment conditions were further compared (**Supplementary Figure 3D**). Statistical analysis demonstrated that the trend of SI values in the rL-TGF-β2-treated group and the LPS/rL-TGF-β2 co-treated group was significantly different compared to the LPS-treated group. These results revealed that L-TGF-β2 could play an inhibitory role in the proliferation of leukocytes under both quiescent as well as activated states, which is different from the previously described bidirectional regulation of SMB cell proliferation by L-TGF-β2.

### The Regulatory Effect of L-TGF-β2 on the Apoptosis of SMB Cells and Leukocytes in the Lamprey

**Figure 7:**
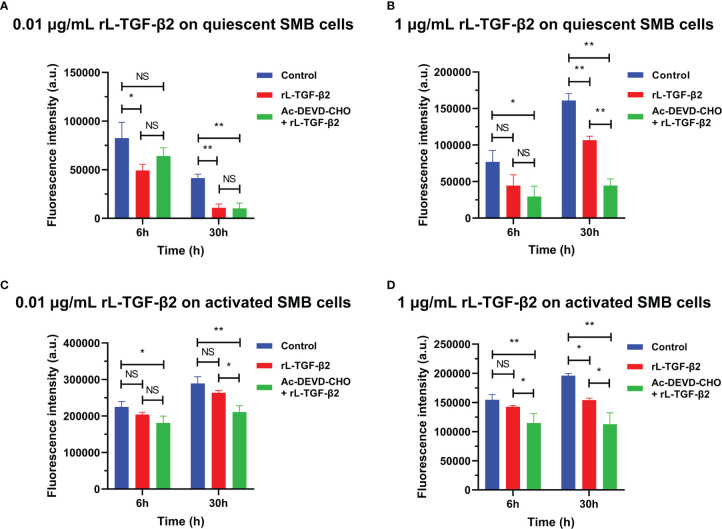
The effect of rL-TGF-β2 on apoptosis of quiescent and activated SMB cells in lampreys detected by quantification of caspase-3/7 activity. After 6 or 30 h of incubation, the fluorescence intensity of the L-TGF-β-treated group, Ac-DEVD-CHO + L-TGF-β co-treated group, and the control group were compared, respectively. All experiments were repeated thrice. The statistical analyses were all performed by ordinary one-way ANOVA followed by Tukey’s multiple comparisons test, in which the statistical significance between the two groups is indicated by the asterisk at the top of the bar chart. *: *p* < 0.05, **: *p* < 0.01, NS, Not significant. **(A)** The effect of rL-TGF-β2 (0.01 ug/ml) on apoptosis of quiescent SMB cells. **(B)** The effect of rL-TGF-β2 (1 ug/ml) on apoptosis of quiescent SMB cells. **(C)** The effect of rL-TGF-β2 (0.01 ug/ml) on apoptosis of activated SMB cells. **(D)** The effect of rL-TGF-β2 (1 ug/ml) on apoptosis of activated SMB cells.

To examine the possible role of lamprey TGF-βs in apoptosis, we first induced apoptosis in primary cultured lamprey SMB cells using serum starvation, then treated the cells with two different concentrations of rL-TGF-β2 for 6 h or 30 h, respectively, followed by quantification of caspase-3/7 activity by adding fluorescent substrates of caspase-3/7, thereby analyzing the apoptosis of the cells. The effect of L-TGF-β2 on the apoptosis of quiescent SMB cells was first measured. SMB cells from unimmunized lampreys treated with a low concentration of rL-TGF-β2 (0.01 ug/mL) were found to have significantly lower caspase-3/7 activity compared to the control after both 6 and 30 h, similar to SMB cells treated with both rL-TGF-β2 and Ac-DEVD-CHO, an inhibitor of caspase-3/7 (**Figure 7A**). And when quiescent SMB cells were treated with a high concentration of rL-TGF-β2 (1 ug/ml) for 30 h, the activity of caspase-3/7 was also significantly reduced, although this activity was still higher than in the Ac-DEVD-CHO and rL-TGF-β2 co-treatment group (**Figure 7B**). Alternatively, to analyze the effect of L-TGF-β2 on apoptosis of activated SMB cells, LPS-immunized lampreys-derived SMB cells were treated with different concentrations of rL-TGF-β2 for 6 and 30 h, respectively. It was found that caspase-3/7 activity was significantly decreased only after 30 h of treatment with a high concentration of rL-TGF-β2 (1 ug/ml) compared to the control (**Figures 7C, D**). Thus, these results revealed that rL-TGF-β2 can inhibit caspase-3/7 activity in quiescent and activated SMB cells, suggesting that the lamprey TGF-β2 can inhibit apoptosis of SMB cells induced by low serum. Similarly, the effect of rL-TGF-β2 on the apoptosis of leukocytes was examined. After leukocytes derived from unimmunized or LPS-immunized lampreys were treated with rL-TGF-β2 (1 ug/ml) for 30 h, respectively, the activity of caspase-3/7 in both quiescent and activated leukocytes was significantly increased compared with the control (**Supplementary Figures 4A, B**). Therefore, these results suggested that the lamprey TGF-β2 can promote apoptosis of leukocytes, which is in contrast to the inhibitory effect of L-TGF-β2 on apoptosis in SMB cells.

## Discussion

The TGF-β superfamily signaling pathway is unique to metazoans, and members of the pathway have been identified in all metazoans studied. The emergence and divergence of the true TGF-β subfamily are more recent, appearing only in deuterostomes (2, 50, 51). As mentioned above, lower deuterostomes such as sea urchins contain only a single member of the TGF-β subfamily, whereas in jawed vertebrates the number of TGF-β members is significantly increased, most likely due to the second round of whole-genome replication (2R) and additional rounds in bony fishes. Thus, how the TGF-β subfamily expanded from a single ancestral gene to multiple family members is still a poorly elucidated question, largely due to the lack of genomic data on other representative species intermediate in the evolutionary history of the TGF-β family, particularly jawless vertebrates. As one of the remaining representatives of the jawless vertebrates in the deuterostomes, preliminary identification of the TGF-β superfamily members of the sea lamprey was previously reported, and only one TGF-β2 homolog was found among 12 superfamily ligands (52). This omission in the identification of family members is due to the fact that the public database of the sea lamprey genome uses a draft of the genome with relatively poor assembly quality and considerable gaps (53). Recently, a 1.06 GB reference genome of the reissner lamprey (*Lethenteron reissneri*) (7) was assembled by PacBio’s third-generation sequencing platform. This genome is the first genomic database with the longest N50 at the chromosomal level in lampreys and has a much higher degree of integrity and continuity than the previously published genomes of the sea lamprey and Japanese lamprey.

In this study, we performed a comprehensive search at the genome-wide level to identify members of the lamprey TGF-β subfamily based on the high-quality reissner lamprey reference genome, combined with the sea lamprey genome and Japanese lamprey transcriptome data. We found that a single TGF-β ancestral gene in lower deuterostomes has diverged into two subfamily members, TGF-β2 and 3, in all three species of lampreys, and they were localized at different chromosomal loci. Phylogenetic tree analysis of 17 representative deuterostomes, including the lamprey, further showed that all TGF-β2 and 3 clustered into a single subtree, suggesting that TGF-β2 and 3 are more closely related to each other than to TGF-β1, consistent with previous reports (54, 55). Meanwhile, both the lamprey TGF-β2 and 3 were located at the bottom of the TGF-β2A and TGF-β3 clusters, respectively. The above results indicated that a common TGF-β ancestral gene has diverged in lampreys, possibly due to gene duplication and chromosomal translocation, and the resulting lamprey TGF-β2 and 3 genes are likely the direct ancestors of TGF-β2 and 3 of jawed vertebrates. Additionally, we found relatively high conservation between lamprey TGF-βs and jawed vertebrate TGF-βs in terms of gene structure, surrounding linkage genes, protein domains, and motifs, also supporting our findings. Notably, earlier studies have suggested that the TGF-β family evolved after the divergence of TGF-β1, followed by the divergence of TGF-β2 from TGF-β3 (3, 6). However, we were unable to identify homologs of TGF-β1 in any species of lamprey. Similarly, in cartilaginous fish such as sharks, only TGF-β2 and 3 are present until TGF-β1~3 appear in coelacanths of the lobe-finned fishes. Therefore, whether the lack of TGF-β1 homologs in lampreys is a consequence of gene loss during evolution or due to a later divergence of TGF-β1 than TGF-β2 and 3 still requires more data to clarify. Furthermore, TGF-β2 with two different gene copies (TGF-β2A and TGF-β2B) was found in reissner lamprey and sea lamprey. TGF-β2 gene replication was initially thought to be unique to bony fishes (56), but the presence of TGF-β2B was later also found in elephant sharks, coelacanths, and pythons, implying that the expansion of TGF-β ligands may be attributed to the second round of whole-genome replication (2R) that occurred early in the evolution of jawed vertebrates or additional whole-genome replication in bony fishes (52, 57). However, the phylogenetic tree in our study revealed that the lamprey TGF-β2B was located at the bottom of the TGF-β2A cluster, yet was more distantly related to TGF-β2B in fish. The syntenic relationship analyses also showed that the neighboring gene environment of lamprey TGF-β2B was more similar to that of jawed vertebrate TGF-β2A, but differed significantly from that of bony fish TGF-β2B. These results indicate that the lamprey TGF-β2B is not a direct ancestor of TGF-β2B in fish, but is more closely related to TGF-β2A in jawed vertebrates.

The key features that distinguish true TGF-β from other TGF-β superfamily members include its ability to make it latent by binding to propeptides and to form the large latent complex by covalently binding LTBP (12). Firstly, we found that the prodomains of lamprey TGF-βs had a relatively conserved cysteine residue pattern, which ensured that the LAP formed by TGF-βs propeptides can dimerize and bind to LTBP through disulfide bonds. These findings suggest that during vertebrate evolution, although the sequences of the prodomains of TGF-βs exhibit considerable low conservation, yet the patterns of their cysteine residues are conserved across species, further confirming that the structural stability of LAP and the ability of LAP to covalently bind LTBP are important for the proper processing, secretion, and activity regulation of mature TGF-βs dimers. Secondly, the process of releasing mature TGF-β ligands from their latent complexes by TGF-β activators is a key trigger of the TGF-β signaling cascade response. Among many possible TGF-β activators, the integrins αVβ6 and αVβ8 are the major activators of TGF-β1 and 3 *in vivo*. For example, a mouse model in which the RGD motif responsible for binding integrins in TGF-β1 was replaced with the RGE sequence had a phenotype highly similar to that of TGF-β1-deficient mice, exhibiting severe tissue inflammation and a lack of Langerhans cells in the epidermis. Since mutant TGF-β proteins are normally secreted, this phenotype suggests that the integrin-binding motif in TGF-β1 is essential for TGF-β to function properly and that the Asp residue in the RGD motif is a key residue required for binding of the integrins (48, 58). Moreover, RGD motifs in TGF-βs have been identified from species that are evolutionarily distant from mammals, such as sea urchin and acorn worm, and the RGD sequence in at least one member of the TGF-β family is conserved among all species previously examined. Therefore, integrins may be among the earliest and strictly conserved activators of TGF-β1/3, which were present in the common ancestor of deuterostomes. Remarkably, our study, however, showed that the lamprey TGF-β3 consistently lacked RGD motifs in the integrin-binding region, implying that the integrin-based activation mechanism may not be as strictly conserved as previously proposed and that the lamprey TGF-β3 may have a unique activation mechanism. Another possibility is that the GGD motif in the C-terminus of the prodomain of lamprey TGF-β3 may serve as a binding site for integrins, as it retains a critical Asp residue, and thus TGF-β3 may be activated by the unidentified integrins.

The ligand domains of lamprey TGF-β2 and 3 showed more than 70% amino acid identity with that of human TGF-β1~3. Such a high level of conservation suggests that the mature TGF-β ligands of the lamprey have similar receptor binding capacity and biological functions to that of higher animals, as reflected by their ability to function correctly in cross-phyla experiments. In this study, we found that the lamprey TGF-β2 recombinant protein could inhibit the growth of mammalian tumor cells *in vitro* in a time- and dose-dependent manner, which is consistent with the function of mammalian TGF-βs (59). Studies based on higher vertebrates have shown that the TGF-β subfamily is not only involved in the regulation of embryonic development but is also an important regulator in the immune response. For the lower deuterostomes, the function of TGF-βs has focused on individual development, but it remains unclear when TGF-βs evolved as an immune regulator (54). As mentioned above, there is some evidence for involving TGF-βs in the regulation of immunity only in a limited species of lower jawed vertebrates, such as bony fishes and amphibians. The TGF-β homolog found in the more ancient cephalochordate amphioxus can induce or inhibit the migration of mouse macrophages RAW 264.7 *in vitro* (20), but further data demonstrating its ability to modulate the immune response of amphioxus itself are still lacking. Thus, uncovering whether TGF-βs are among the most ancestral immune cytokines in vertebrates and elucidating the functional evolution of the TGF-β subfamily depends on the research of the lamprey.

Here, our study revealed the transcriptional expression patterns of L-TGF-βs in various tissues of the Japanese lamprey and the changes in their expression levels during the LPS-induced innate immune response. Since our screened antibodies specific for L-TGF-βs were not suitable for immunoblot analysis, we have instead used these antibodies to develop a quantitative sandwich ELISA kit for determining the level of L-TGF-βs proteins. As shown in **Figure 4D**, the total amount of L-TGF-βs including the latent and activated forms secreted by SMB cells were increased significantly after *in vitro* LPS stimulation. Additionally, we also analyzed L-TGFBR1 and L-TGFBR2, which are two types of cell surface receptors for TGF-β in the Japanese lamprey, and found that their expression in SMB cells and leukocytes was also rapidly up-regulated after LPS stimulation (data not shown). These results indicate that the TGF-β signaling pathway contributes to the immune regulation of lamprey. Furthermore, after comparing the response kinetics of L-TGF-β2 and 3 to LPS stimulation, we proposed that L-TGF-β2 may play a central role in the regulation of the innate immune response of the lamprey since it exhibited a more rapid and substantial upregulation of expression than L-TGF-β3.

As multifunctional cytokines, TGF-βs can exert immunomodulatory effects by influencing the proliferation and apoptosis of T and B cells as well as monocytes and macrophages (60–63). Among these functions, the cytostatic effect of TGF-βs is one of their widely studied functions, and numerous studies have shown that TGF-βs can induce cell cycle arrest in various cultured mammalian cells. For example, TGF-β was found to inhibit IL-2-dependent T cell proliferation in humans (64) and to inhibit the proliferation of mature B cells *in vitro* by mediating cell cycle arrest (G1/S transition) (65). In this study, to analyze the possible role of lamprey TGF-βs in immune regulation, SMB cells and peripheral blood leukocytes in the Japanese lamprey were stimulated with recombinant L-TGF-β2 mature peptide, and cell proliferation was measured using an ELISA-based detection of the incorporation of BrdU assay, which is a sensitive indicator of mitogen response. The BrdU method has advantages over other colorimetric methods that are strongly influenced by the metabolic state of leukocytes and has been applied to the detection of mitosis of leukocytes *in vitro* in several species of bony fish (23, 66). Significantly, our study revealed for the first time that rL-TGF-β2 can exert the bipolar regulation on the proliferation of quiescent and activated SMB cells, which play an important role in the immune response of lampreys (33, 67, 68). In comparison to SMB cells, L-TGF-β2 can inhibit the proliferation of leukocytes under both quiescent and activated states. Additionally, we noted several differences in the response kinetics between SMB cells and leukocytes to L-TGF-β2 stimulation. For example, L-TGF-β2 treatment of quiescent and activated leukocytes exhibited a significant anti-proliferative effect at 24 h (**Supplementary Figures 3B, C**). However, SMB cells responded more slowly to L-TGF-β2 than leukocytes. L-TGF-β2 did not significantly promote the proliferation of quiescent SMB cells until 96 h after treatment, whereas the BrdU incorporation from 24 to 72 h after L-TGF-β2 treatment was not statistically different from that of the controls, although there was a trend toward upregulation (**Figure 6B**). Especially in activated SMB cells, L-TGF-β2 first significantly promoted cell proliferation after 24 and 48 h of treatment, and the anti-proliferative effect of L-TGF-β2 on activated SMB cells appeared from 72 h after treatment (**Figure 6C**). The characteristic delayed response of SMB cells to L-TGF-β2 may be due to the possibility that the regulation of the cell cycle by the TGF-β signaling pathway differs between SMB cells and leukocytes, and its molecular mechanism deserves in-depth investigation. It is found that the role of TGF-βs in the immune system is context-dependent in bony fishes and mammals, where the type of target cell, differentiation status, and other regulatory signals can influence the cellular response to TGF-β signal, and the bipolar function of TGF-β proteins is of great importance for maintaining immune homeostasis (1, 24, 25, 43, 69). Here, our study reveals that such a context-dependent dynamic function of TGF-βs can be tracked evolutionarily back to the primitive jawless vertebrate, the lamprey, and suggests that L-TGF-β2 serves as the key immunomodulatory molecule in maintaining the homeostasis of the immune system of the lamprey.

Furthermore, the regulation of cell proliferation is only one aspect of the multiple biological effects of TGF-βs. TGF-βs can also induce or inhibit programmed cell death in different cell types. For example, TGF-β-mediated apoptosis plays a key role in maintaining homeostasis of B and T cells in mammals (70). In this study, early apoptosis was detected and quantified by adding a fluorescent substrate of caspase-3/7 to lamprey SMB cells or leukocytes under serum deprivation conditions, and then analyzed whether L-TGF-β2 can affect apoptosis during the immune response in lampreys. Caspase-3 and 7 are the main executors of apoptosis and are involved in the delivery of apoptotic signals. Among all caspase family members, caspase-7 was reported to be widely and highly expressed in various tissues of lampreys, in contrast to the lamprey caspase-3 gene which was expressed at the lowest level in all tissues (71), implying that caspase-7 may play a more important role in lampreys compared to caspase-3. Consistent with previous studies (71), we also found that specific inhibitors of human caspase-3/7 effectively inhibited apoptosis in lampreys. Notably, we found for the first time that recombinant L-TGF-β2 inhibited serum deprivation-induced caspase-7 activity in SMB cells but promoted caspase-7 activity in leukocytes, and this effect was independent of whether SMB cells and leukocytes were in a quiescent or activated state. Our results suggest that lamprey TGF-β2 suppresses early apoptosis of SMB cells but enhances early apoptosis of leukocytes. Based on numerous studies in mammals, TGF-βs were considered as potent inducers of lymphocyte apoptosis in the immune system (72), but in some cases, TGF-βs can also inhibit apoptosis. For example, in both follicular dendritic cells (73) and microglia (74), TGF-βs could prevent Fas-mediated programmed cell death. Additionally, TGF-βs can protect macrophages from apoptosis after serum deprivation (75). Therefore, our study reveals that the promotion or suppression of apoptosis by TGF-βs in lampreys is cell type dependent, as in mammals (59).

In conclusion, our study not only offers valuable clues to the origin and evolution of the TGF-β subfamily in vertebrates but also provides a basis for understanding the different roles of TGF-βs in regulating cell proliferation and survival in the immune system of the lamprey. All these results highlight that TGF-βs may be one of the most ancient immunomodulatory molecules in vertebrates, and their exact functions and underlying mechanisms in maintaining immune homeostasis in lampreys are still under intensive investigation.

## Data Availability Statement

The original contributions presented in the study are included in the article/**Supplementary Material**, further inquiries can be directed to the corresponding authors.

## Ethics Statement

The animal study was reviewed and approved by Animal Welfare and Research Ethics Committee of the Institute of Dalian Medical University. Written informed consent was obtained from the owners for the participation of their animals in this study.

## Author Contributions

HW and QL designed the experiments. Molecular evolution analyses and apoptosis assay were finished by SQL. Gene cloning and real-time PCR were finished by JG. Recombinant protein expression and proliferation assay in SMB cells were finished by XDC. ELISA and proliferation assay in leukocytes were finished by WL. Cell culture was finished by SYL and XYC. HW analyzed the data and wrote the first draft of the manuscript. SQL, JG, XDC, and WL wrote sections of the manuscript. All authors contributed to manuscript revision, read, and approved the submitted version.

## Funding

This work was supported by the National Natural Science Foundation of China [grant number 31601150], the Postdoctoral Science Foundation of China [grant number 2016M601329], and the Doctoral Start-up Foundation of Liaoning Province [grant number 201601241].

## Conflict of Interest

The authors declare that the research was conducted in the absence of any commercial or financial relationships that could be construed as a potential conflict of interest.

## Publisher’s Note

All claims expressed in this article are solely those of the authors and do not necessarily represent those of their affiliated organizations, or those of the publisher, the editors and the reviewers. Any product that may be evaluated in this article, or claim that may be made by its manufacturer, is not guaranteed or endorsed by the publisher.
