# Supplementary material for: Molecular Evolution of Transforming Growth Factor-β (TGF-β) Gene Family and the Functional Characterization of Lamprey TGF-β2
Source: Front Immunol. 2022 Mar 4;13:836226. doi: 10.3389/fimmu.2022.836226 (PMC8931421; doi:10.3389/fimmu.2022.836226)
Supplement: Supplementary file 1 [file DataSheet_1.docx]

Supplementary Material

**Supplementary Table 1. The TGF-β protein accession numbers in all examined species**

| **Species** | **Symbol** | **Accession No.** | **Species** | **Symbol** | **Accession No.** |
| --- | --- | --- | --- | --- | --- |
| **Zebrafish**  ***(Danio rerio*)** | TGFB1A | NP_878293.1 | **Burmese python**  **(*Python bivittatus*)** | TGFB2B | XP_007434440.1 |
|  | TGFB1B | XP_692338.3 |  | TGFB3 | XP_007432369.1 |
|  | TGFB2A | NP_919366.1 | **Coelacanth**  **(*Latimeria chalumnae*)** | TGFB1 | XP_005987924.1 |
|  | TGFB2B | XP_688180.1 |  | TGFB2A | XP_005998998.1 |
|  | TGFB3A | NP_919367.2 |  | TGFB2B | XP_005988913.1 |
| **Vase tunicate**  **(*Ciona intestinalis*)** | TGFB | NP_001071838.1 |  | TGFB3 | XP_005986600.1 |
| **Acorn Worm**  **（*Saccoglossus kowalevskii*）** | TGFB2 | NP_001171727.1 | **Western clawed frog**  **(*Xenopus tropicalis*)** | TGFB1  TGFB2 | XP_002939433.1  XP_002936067.1 |
| **Purple sea urchin**  **（*Strongylocentrotus purpuratus*）** | TGFB2 | XP_793246.2 | **Human**  **(*Homo sapiens*)** | TGFB1  TGFB2  TGFB3 | NP_000651.3  NP_003229.1  NP_003230.1 |
| **Chicken**  **(*Gallus gallus*)** | TGFB1 | NP_001305385.1 |  |  |  |
|  | TGFB2 | NP_001026216.2 |  |  |  |
|  | TGFB3 | NP_990785.1 | **Elephant shark**  **(*Callorhinchus milii*)** | TGFB2A  TGFB2B | XP_007902706.1  XP_007883723.1 |
| **Nile tilapia (*Oreochromis niloticus*)** | TGFB1B  TGFB2A  TGFB2B  TGFB3A  TGFB3B | XP_003459502.1  XP_005464049.1  XP_003444651.1  XP_003453117.1  XP_003455646.1 |  |  |  |
|  |  |  | **Whale shark**  **(Rhincodon typus)** | TGFB3 | XP_020376834.1 |
|  |  |  | **Mouse**  **(*Mus musculus*)** | TGFB1  TGFB2  TGFB3  GDNF | NP_035707.1  NP_001316036.1  NP_033394.2  NP_034405.1 |
| **Green anole**  **(*Anolis carolinensis*)** | TGFB1  TGFB3 | XP_003222953.1  XP_003214485.1 |  |  |  |
|  |  |  | **Sea lamprey**  **(*Petromyzon marinus*)** | TGFB2A  TGFB2B  TGFB3 | XP_032818531.1  XP_032803552.1  XP_032821066.1 |
| **Common lizard**  **（*Zootoca vivipara*）** | TGFB2 | XP_034967246.1 |  |  |  |
| **Burmese python**  **(Python bivittatus)** | TGFB1  TGFB2A | XP_007421553.1  XP_015742830.2 |  |  |  |

**Supplementary Table 2. The matching amino acid sequences in motifs**

| **Motif** | **Lenth (nt)** | **Best possible match** |
| --- | --- | --- |
| Motif 1 | 50 | YIDFRRDLGWKWIHEPKGYYANFCAGPCPYLWSADTQHSKVLSLYNTJNP |
| Motif 2 | 41 | ASASPCCVPQDLEPLTILYYVGRTPKVEQLSNMVVKSCKCS |
| Motif 3 | 29 | STCSTLDMDHIKRKRIEAIRGQILSKLKL |
| Motif 4 | 29 | WLLHRDRNLGLKISVHCPCCTFVPSNNII |
| Motif 5 | 50 | GPYFRIFRFBVSSMEKNASNLVKAEFRVFRLPNPKARVSEQRIELYQILK |
| Motif 6 | 21 | YJDSKVVRTRAEGEWLSFDVT |
| Motif 7 | 21 | KKRALDTAYCFRNVEDNCCLR |
| Motif 8 | 21 | PEEVPPZVLALYNSTRELLZE |
| Motif 9 | 21 | CERERSEEEYYAKEVHKFDMJ |
| Motif 10 | 21 | TPHLJLMLLPPYRLESQQSSR |
| Motif 11 | 15 | PNKSEELEARFAGID |
| Motif 12 | 29 | IYFFFNVSEIRENVPDPKLLYRAELRMRR |
| Motif 13 | 8 | DPTSPKQR |
| Motif 14 | 15 | LVFLLLNLATVALSL |
| Motif 15 | 15 | DEHGRGDLGRLKKQK |
| Motif 16 | 8 | TSPPEDYG |
| Motif 17 | 8 | KANERAAT |
| Motif 18 | 11 | PEHNELPYCPK |
| Motif 19 | 41 | APADSCKCGGGGFHGEVGHCSQATFSKGIPRGKDKAPQISH |
| Motif 20 | 20 | KRRHERQQQQQHHQHGGAWD |

**Supplementary Table 3. List of retrieved genes data from Genomicus in the syntenic analyses**

| **Species** | **Symbol** | **Accession No** |
| --- | --- | --- |
| Human | TGFB2 | ENSG00000092969 |
| Mouse | TGFB2 | ENSMUSG00000039239 |
| Zebrafish | TGFB2A | ENSDARG00000027087 |
| Zebrafish | TGFB2B | ENSDARG00000071879 |
| Coelacanth | TGFB2A | ENSLACG00000006847 |
| Coelacanth | TGFB2B | ENSLACG00000017213 |
| Human | TGFB3 | ENSG00000119699 |
| Mouse | TGFB3 | ENSMUSG00000021253 |
| Zebrafish | TGFB3 | ENSDARG00000019367 |
| Coelacanth | TGFB3 | ENSLACG00000016642 |

**Supplementary Table 4. List of primer sequences for gene cloning and qPCR**

| **Gene** | **Primer** | **Sequences (5’ to 3')** | **Application** |
| --- | --- | --- | --- |
| L-TGF-β2 | Forward | ATGCATTGTCGCAGCCTC | gene cloning |
|  | Reverse | CTAGCTGCACTTGCAGGA |  |
| L-TGF-β3 | Forward | TGAATTCTCCGTGTGACAGC | gene cloning |
|  | Reverse | TCCGGTAATCTAACTCGGGTC |  |
| L-TGF-β2 | Forward | CGCCAAGGAGGTCCACAT | qPCR |
|  | Reverse | TCGGAAGTAGTCGTTGTAAGGAT |  |
| L-TGF-β3 | Forward | ACGGGCAGTCGGAGGAGAAC | qPCR |
|  | Reverse | GTCGAGCCAGAGCAGCAAG |  |
| Gapdh | Forward | AACCAACTGCCTGGCTCCT | qPCR |
|  | Reverse | GTCTTCTGCGTTGCCGTGT |  |


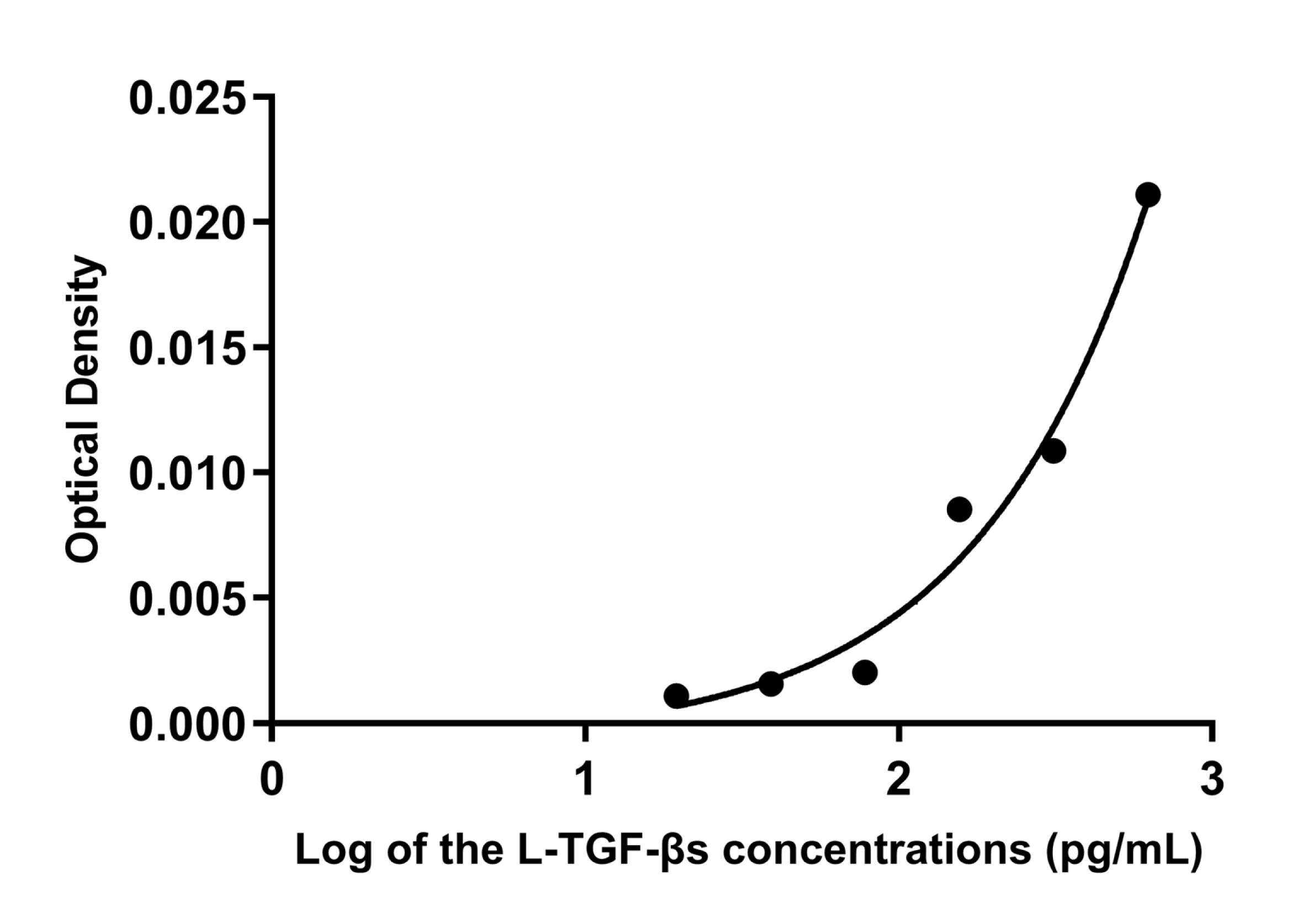


**Supplementary Figure 1.** The standard curve of L-TGF-β concentration in ELISA. Based on a customized sandwich ELISA kit, the standard curve for the quantification of TGF-β proteins in lampreys was plotted using the four-parameter logistic (4-PL) curve-fit method with different concentrations of recombinant L-TGF-β2 protein as standards. R^2^ = 0.9763.


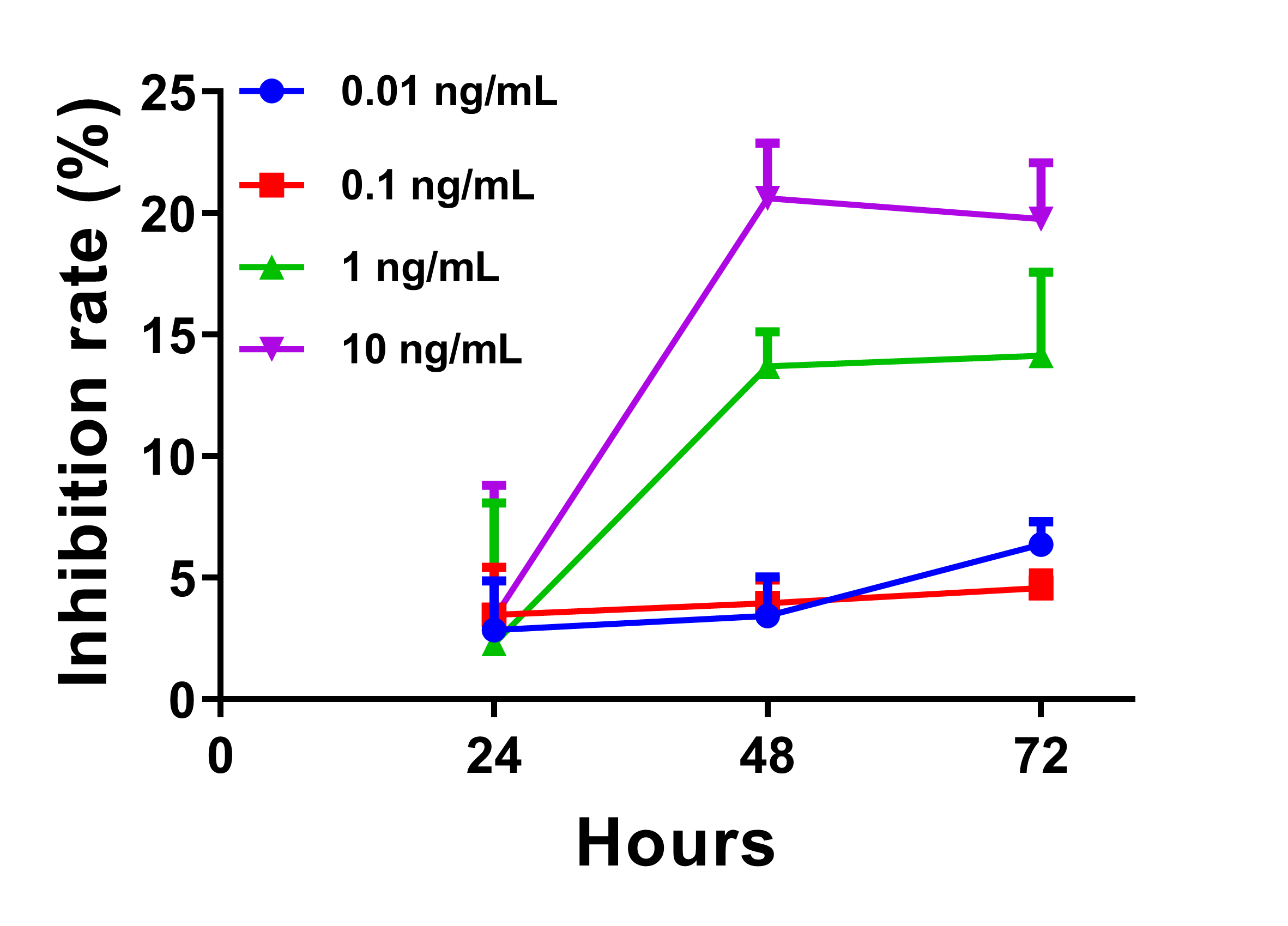


**Supplementary Figure 2.** The inhibition rate of human TGF-β2 on the proliferation of MCF-7 cells using CCK8 assay. Two-way RM ANOVA is used for statistical analysis. Time: F (1.310, 10.48) = 7.563, *P* = 0.0150; dose: F (3, 8) = 23.55, *P* = 0.0003; the interaction of time and dose: F (6, 16) = 1.875, *P* = 0.1475.


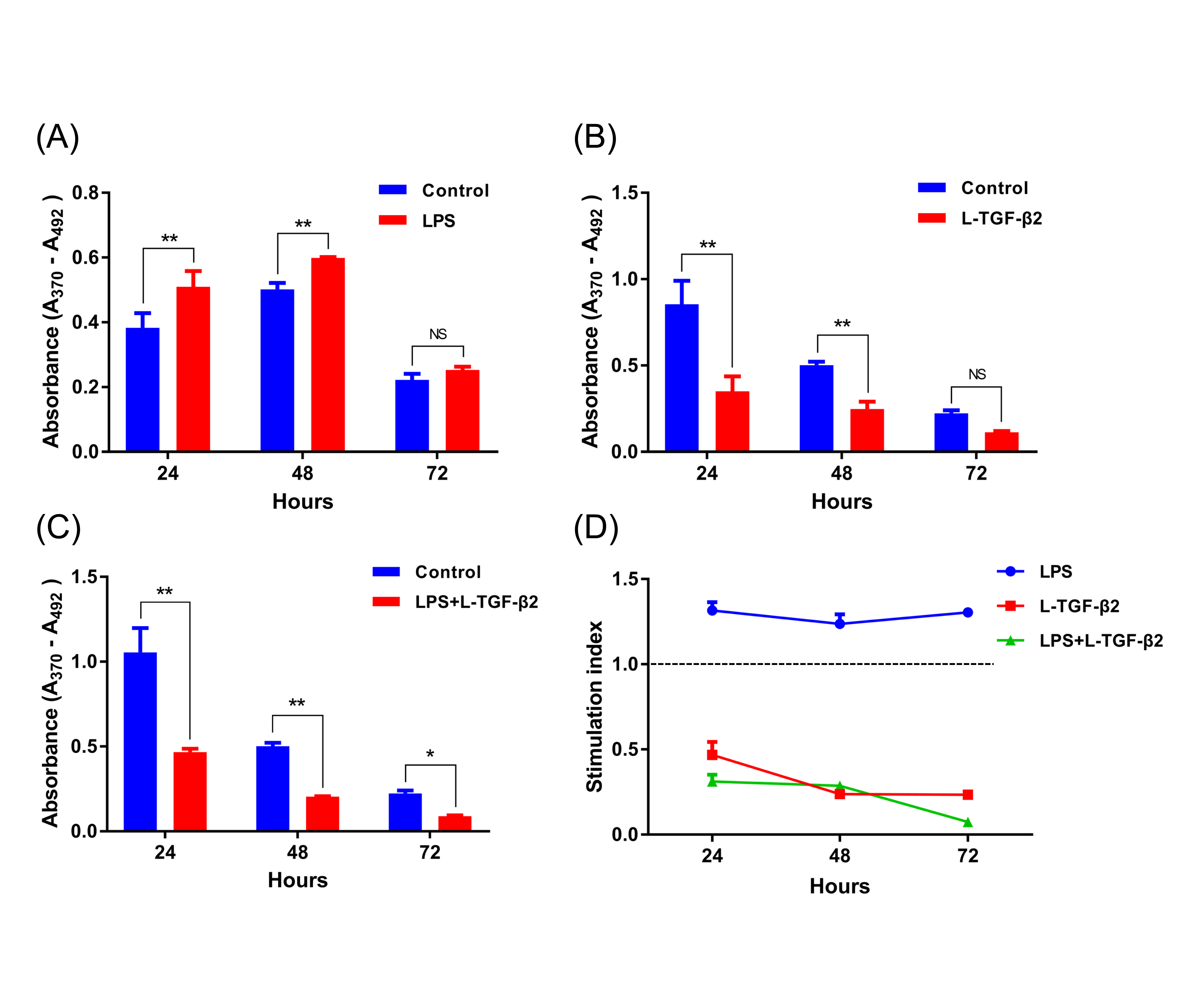


**Supplementary Figure 3.** The effect of rL-TGF-β2 on proliferation of quiescent and activated peripheral blood leukocytes in lampreys. **(A)** The effect of LPS on the proliferation of leukocytes. The OD values are compared between the LPS-treated groups with the control after 24, 48, and 72 h of incubation. **(B)** The effect of rL-TGF-β2 on the proliferation of quiescent leukocytes. The OD values are compared between the rL-TGF-β2-treated group with the control after 24 to 72 h of incubation. **(C)** The effect of rL-TGF-β2 on the proliferation of LPS-stimulated leukocytes. The OD values are compared between the rL-TGF-β2-treated group with the control after 24 to 72 h of incubation. In Figures A to C, the statistical analyses are all performed by multiple *t*-tests, in which the statistical significance is determined using the Holm-Sidak method, with alpha = 0.05. *: Adjusted *p* < 0.05, **: Adjusted *p* < 0.01. All experiments were repeated thrice. **(D)** Trends in the stimulation index of leukocytes proliferation under different treatment conditions. Two-way RM ANOVA is used for statistical analysis. Treatment condition: F (2, 6) = 1492, *P* < 0.001; Treatment time: F (2, 12) = 40.23, *P* < 0.001; Interaction: F (4, 12) = 16.33, *P* < 0.001. The LPS-treated group was subsequently set as the control for Dunnett's multiple comparison test. LPS-treated group vs. rL-TGF-β2-treated group and LPS-treated group vs. LPS + rL-TGF-β2 co-treated group were significantly different at all time points, *P* < 0.001.


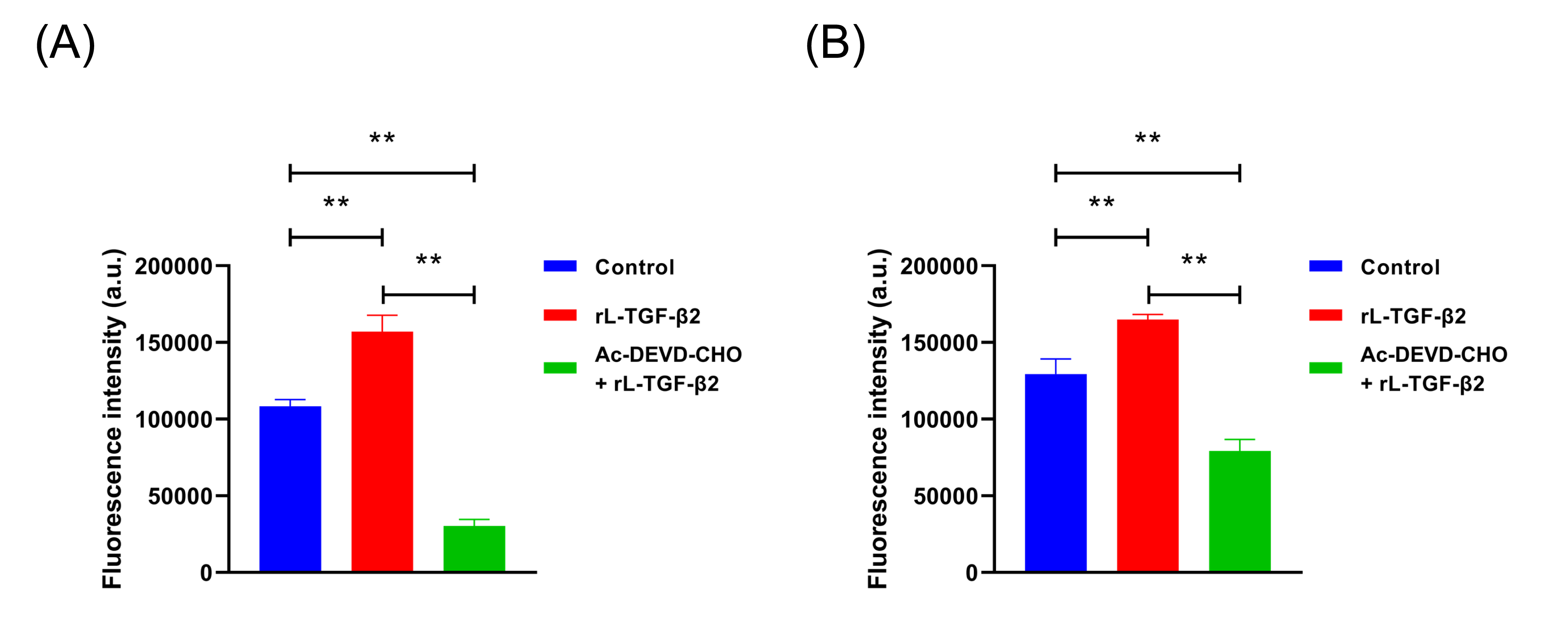


**Supplementary Figure 4.** The effect of rL-TGF-β2 on apoptosis of quiescent and activated peripheral blood leukocytes in lampreys detected by quantification of caspase-3/7 activity. After 30 h of incubation, the fluorescence intensity of the rL-TGF-β2-treated group, Ac-DEVD-CHO + rL-TGF-β2 co-treated group, and the control group were compared, respectively. All experiments were repeated thrice. *: p < 0.05, **: p < 0.01. (A) The effect of rL-TGF-β2 (1 ug/ml) on apoptosis of quiescent leukocytes. (B) The effect of rL-TGF-β2 (1 ug/ml) on apoptosis of activated leukocytes.
